# Arthrocolins Synergizing with Fluconazole Inhibit Fluconazole-Resistant Candida albicans by Increasing Riboflavin Metabolism and Causing Mitochondrial Dysfunction and Autophagy

**DOI:** 10.1128/spectrum.04051-22

**Published:** 2023-02-27

**Authors:** Zhuang Wu, Qun-Fu Wu, Wen-Li Yuan, Yong-Hong Chen, Di Hu, De-Yao Deng, Long-Long Zhang, Xue-Mei Niu

**Affiliations:** a Laboratory for Conservation and Utilization of Bio-Resources, Key Laboratory for Microbial Resources of the Ministry of Education, Yunnan University, Kunming, People’s Republic of China; b Department of Clinical Laboratory, The Affiliated Hospital of Yunnan University, The second hospital of Yunnan Province, Kunming, Yunnan Province, People’s Republic of China; Agricultural Research Organization, Volcani Center

**Keywords:** arthrocolins, fluconazole-resistant *Candida albicans*, membrane transports, mitochondrial dysfunctions, proteasomes, autophagy, riboflavin metabolism

## Abstract

Our previous study reported that seminaturally occurring arthrocolins A to C with unprecedented carbon skeletons could restore the antifungal activity of fluconazole against fluconazole-resistant Candida albicans. Here, we showed that arthrocolins synergized with fluconazole, reducing the fluconazole minimum and dramatically augmenting the survivals of 293T human cells and nematode Caenorhabditis elegans infected with fluconazole-resistant C. albicans. Mechanistically, fluconazole can induce fungal membrane permeability to arthrocolins, leading to the intracellular arthrocolins that were critical to the antifungal activity of the combination therapy by inducing abnormal cell membranes and mitochondrial dysfunctions in the fungus. Transcriptomics and reverse transcription-quantitative PCR (qRT-PCR) analysis indicated that the intracellular arthrocolins induced the strongest upregulated genes that were involved in membrane transports while the downregulated genes were responsible for fungal pathogenesis. Moreover, riboflavin metabolism and proteasomes were the most upregulated pathways, which were accompanied by inhibition of protein biosynthesis and increased levels of reactive oxygen species (ROS), lipids, and autophagy. Our results suggested that arthrocolins should be a novel class of synergistic antifungal compounds by inducing mitochondrial dysfunctions in combination with fluconazole and provided a new perspective for the design of new bioactive antifungal compounds with potential pharmacological properties.

**IMPORTANCE** The prevalence of antifungal-resistant Candida albicans, which is a common human fungal pathogen causing life-threatening systemic infections, has become a challenge in the treatment of fungal infections. Arthrocolins are a new type of xanthene obtained from Escherichia coli fed with a key fungal precursor toluquinol. Different from those artificially synthesized xanthenes used as important medications, arthrocolins can synergize with fluconazole against fluconazole-resistant Candida albicans. Fluconazole can induce the fungal permeability of arthrocolins into fungal cells, and then the intracellular arthrocolins exerted detrimental effects on the fungus by inducing fungal mitochondrial dysfunctions, leading to dramatically reduced fungal pathogenicity. Importantly, the combination of arthrocolins and fluconazole are effective against C. albicans in two models, including human cell line 293T and nematode Caenorhabditis elegans. Arthrocolins should be a novel class of antifungal compounds with potential pharmacological properties.

## INTRODUCTION

Human pathogenic fungi infect billions of people worldwide and kill individuals in excess of 1.5 million per year (1–3). Over the past several decades, conventional antifungal medications are becoming increasingly ineffective at combating infectious disease due to increasing drug-resistant strains (4). Among them, Candida albicans is the most common fungal species isolated from biofilms either formed on human tissue or on implanted medical devices. A mortality rate of 40% has been reported for patients with C. albicans infection. Multiple resistance mechanisms have been reported for C. albicans to counteract antifungal agents, particularly the azole drugs (5). As a widely applicable azole, fluconazole (FLC) can effectively inhibit fungal growth by preventing the biosynthesis of ergosterol, the major sterol component of fungal plasma membranes, leading to increased fluidity of the membrane and altered activity of several membrane-bound enzymes (6). However, fungal resistance to this antifungal medication is increasing (7). There is an urgent need to identify effective antifungal compounds with new mechanisms of action (8–11). The discovery of novel antifungal compounds with distinct structures and impressive biological activities is an approach for the development of new therapeutic strategies for such drug-resistant strains (12–15).

**FIG 1:**
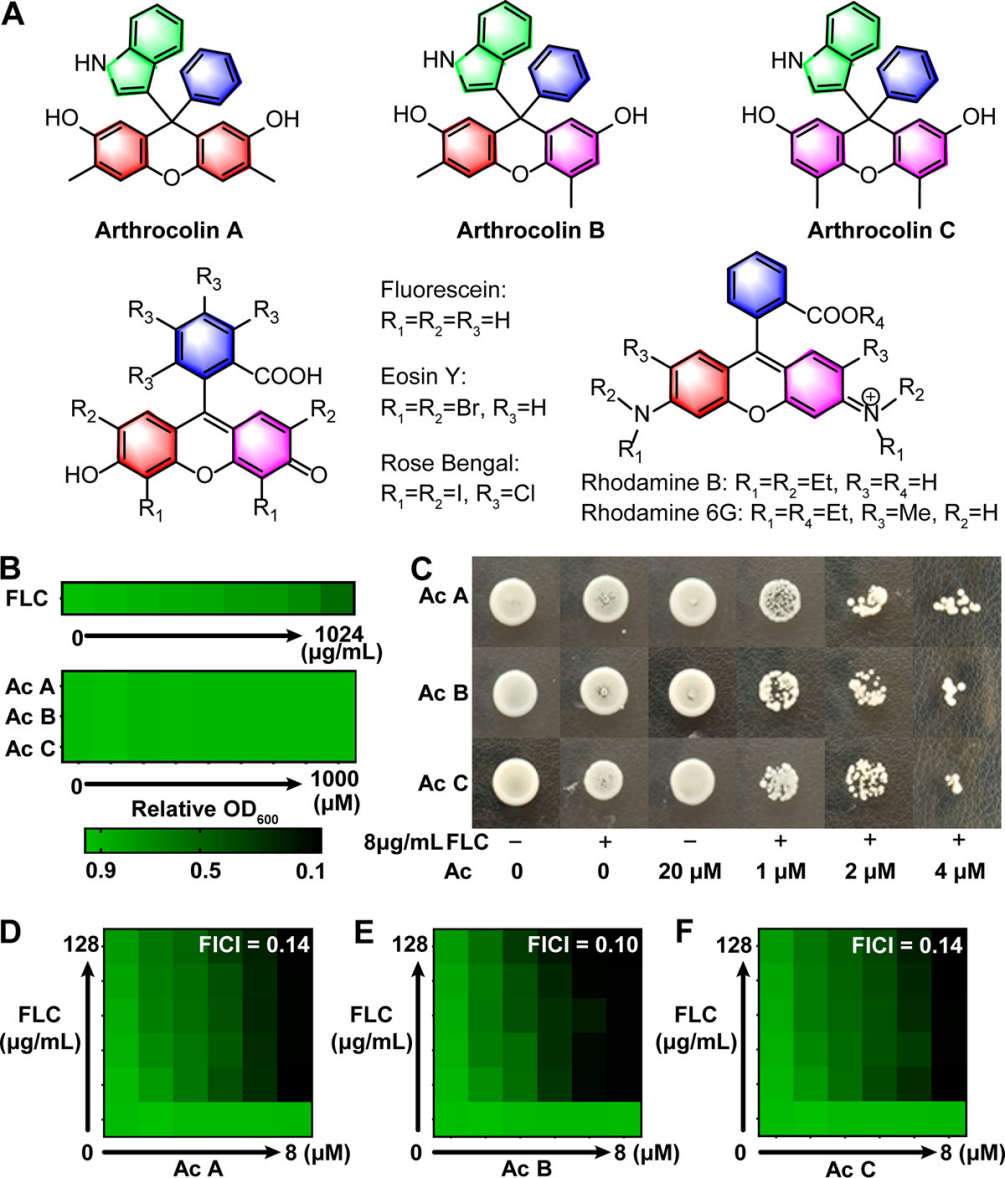
(A) The structures of Acs and the representative fluorescent dyes including fluorescein, rhodamines, and eosins. (B to F) The strong inhibitory effects of Acs A to C synergizing with FLC on the growth of FLC-resistant C. albicans. (B) Checkerboard assays were performed to evaluate the effects of FLC (0 to 1,024 μg/mL, 2-fold dilution series) or Acs A to C (0 to 1,000 μM, 2-fold dilution series) on the growth of FLC-resistant C. albicans. OD_600_ value indicates the fungal growth. Growth in each well is presented in heat-map format based on the OD_600_ of wells at 24 h relative to the no-drug control (see color bar). (C) Spot assays were carried out to evaluate the effects of Ac+FLC, Ac (1, 2, 4, and 20 μM) in combination with FLC (8 μg/mL), on the growth of FLC-resistant C. albicans. (D to F) The strong synergy effects of Acs A to C with FLC on inhibiting FLC-resistant C. albicans. Acs (0, 0.5, 1.0, 2.0, 4.0, and 8.0 μM) and FLC (0, 8, 16, 32, 64, and 128 μg/mL). Ac A, arthrocolin A (D); Ac B, arthrocolin B (E); Ac C, arthrocolin C (F). The fractional inhibitory concentration index (FICI) was calculated to assess interaction effect with a value of <0.5 indicating synergy.

In our continuing research on natural products with antifungal activities from microbes, we found that arthrocolins (Acs) A to C (Fig. 1A) obtained from Escherichia coli fed with a key precursor, toluquinol, that was widely distributed in many fungi, could immensely recover the antifungal activity of FLC against intractable FLC-resistant C. albicans (16). Different from artificially synthesized fluorescent xanthenes, Acs are a class of seminaturally occurring fluorescent dye-like xanthenes with an unprecedented carbon skeleton containing a rare indolyltriphenyl quaternary carbon. The traditionally artificially synthesized xanthenes are an important class of heterocyclic condensed aromatics with an impressive range of pharmacological, industrial, and synthetic applications, including well-known fluorescent dyes such as fluorescein, rhodamines, and eosins (17, 18). They have been widely used as one of the most important medications needed in a basic health system on the World Health Organization’s List of Essential Medicines (18). However, study of the fluorescent dye-like complex xanthenes against human pathogen fungi is still lacking.

The underlying mechanism of Acs in combination with FLC against the FLC-resistant C. albicans is still unknown. In this study, we first observed that the combination of Acs with FLC caused a major reduction in the growth of the FLC-resistant C. albicans both *in vitro* and *in vivo* at low concentrations. Importantly, Acs in combination with FLC could induce fungal membrane permeability to Acs and result in subsequent inhibition of fungal growth via the depletion of intact mitochondria. Interestingly, we found that Acs played a paramount role in upregulating riboflavin metabolism and inducing mitochondrial dysfunction, which caused increased reactive oxygen species (ROS), lipid, and autophagy levels, eventually leading to fungal death.

## RESULTS

### Acs functioned synergistically with antifungal FLC against FLC-resistant C. albicans.

Two human C. albicans strains, one FLC-sensitive strain and one FLC-resistant strain, were used to evaluate the effects of FLC and Acs on fungal growth. Our study displayed that FLC at a concentration of 4 μg/mL could inhibit the FLC-sensitive strain (MIC_90_ value in yeast extract-peptone-dextrose [YPD] medium or RPMI medium, 4 μg/mL) while the FLC-resistant C. albicans strain displayed strong resistance to FLC even at a concentration of 1,024 μg/mL (MIC_90_, >1,024 μg/mL) (Fig. 1B and C). None of the three Ac compounds at concentrations of 0.5 to 1,000 μM displayed any inhibitory activities toward either the FLC-sensitive and FLC-resistant C. albicans strains (MIC, >1,000 μM) (Fig. 1B and C; see also Fig. S1 in the supplemental material). Interestingly, in the presence of FLC at a concentration of 1 μg/mL, Acs at concentrations of 0.5 to 8 μM displayed strong inhibition toward the FLC-sensitive C. albicans. Furthermore, in the presence of FLC at concentrations of 8 to 128 μg/mL, all of the Acs at the concentrations of 0.5 to 8 μM displayed strong inhibition toward the FLC-resistant C. albicans strain (Fig. 1B and C). Further experimentation to evaluate the fractional inhibitory concentration index at 90% growth inhibition (FICI_90_) for the combination of Acs (0.5 to 8 μM) and FLC (8 to 128 μg/mL) revealed results of 0.14, 0.10, and 0.14 for Acs A to C, respectively (Fig. 1D to F; see also Table S1 in the supplemental material), suggesting a strong synergistic interaction between Acs and FLC because FICI_90_ values of <0.5 indicate a synergistic interaction (19, 20). Interestingly, when the concentrations of fluconazole were constant at 1 to 8 μg/mL, the inhibitory effects of Ac+FLC on the FLC-resistant C. albicans strain were dependent on the concentrations of the Acs. However, when the concentration of the Acs was constant at 2 to 8 μM, the inhibition rates of the combination were not related to the concentrations of FLC.

### Combination of Ac and FLC impaired pathogenicity of FLC-resistant C. albicans in 293T cells.

**FIG 2:**
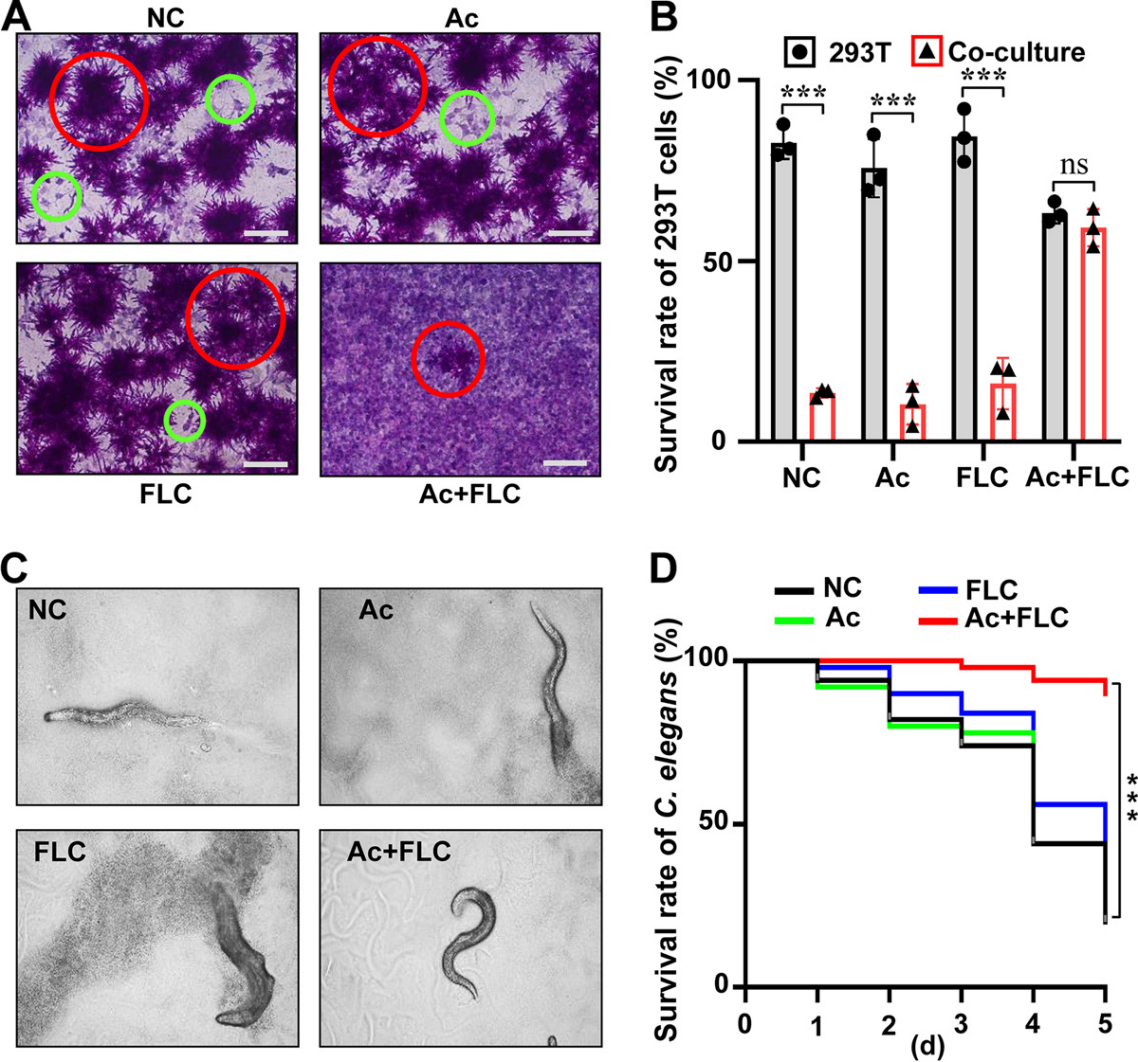
The combination of Ac+FLC impaired the pathogenicity of FLC-resistant C. albicans *in vivo*. NC, DMSO only as negative control; Ac, arthrocolins; FLC, fluconazole. (A) The increased survival of 293T cells infected with FLC-resistant C. albicans after treatment with Ac+FLC. The visualized fungal burden and 293T cells with PAS staining. Red circles refer to FLC-resistant C. albicans; green circles refer to 293T cells. Scale bar, 100 μm. (B) The survival rates of 293T cells cultured without (gray column) and with FLC-resistant C. albicans (red column) under different drug treatments. ***, *P *< 0.001, two-tailed Student's *t* test. Data represent mean ± SD from three independent biological replicates. ns, no significant difference (*P *> 0.05). (C and D) The combination of Ac+FLC dramatically prolonged the life span of the C. elegans that were infected by C. albicans. ***, *P < *0.001, two-way ANOVA.

We investigated the therapeutic potential of the combination of Ac with FLC in human embryonic kidney 293T cells infected with FLC-resistant C. albicans as described in the literature (21). Human 293T cells were cocultured with the FLC-resistant C. albicans at the concentration of 2.5 × 10^3^ per well. Then, 293T cells in coculture with FLC-resistant C. albicans were treated with (i) the combination of 1.0 μM Ac plus 8 μg/mL FLC (Ac+FLC), (ii) 2.0 μM Ac, (iii) 8 μg/mL FLC, and (iv) solvent alone. Ac only and FLC only were used as two controls and solvent as negative control (NC). After 48 h, the survival rates of 293T cells were measured for the evaluation of fungal pathogenicity (22). In NC, Ac, or FLC group, most of the 293T cells were dead with abundant fungal colonies of the FLC-resistant C. albicans strain. However, in the Ac+FLC group, most of the 293T cells remained alive with few colonies of the FLC-resistant C. albicans strain (Fig. 2A). These results suggested that the combination of Ac+FLC dramatically inhibited fungal growth and pathogenicity and increased the survival of 293T human cells infected with FLC-resistant C. albicans. Further analysis with periodic acid-Schiff (PAS) staining of polysaccharides to visualize the effects of the various treatments on both the fungal and human cell elements within the cocultures was performed. We observed extensive damage to the human cell monolayer (stained pale purple) of 293T cells with the abundant FLC-resistant C. albicans strain (stained red) in the Ac, FLC, and NC groups. However, in the Ac+FLC group, most of the 293T cells treated with FLC-resistant C. albicans survived with an intact human cell monolayer (Fig. 2B). This confirmed that the Ac+FLC combination was effective at reducing fungal growth and pathogenicity toward 293T cells.

### Combination of Ac+FLC dramatically increased survival of nematodes infected with FLC-resistant C. albicans.

We then tested the Ac+FLC efficacy *in vivo* using Caenorhabditis elegans as an infectious model. Nematodes were infected with a dose of the FLC-resistant C. albicans that gradually killed the larvae over a period of 5 days. Two hours postinoculation, 50 age-matched L1 stage worms were treated with Ac, FLC, Ac+FLC, and solvent, respectively. The survival rates of the worms were monitored daily. The survival of nematodes treated with Ac+FLC (90.0%) was significantly higher than that of those treated with either drug alone (FLC, 38.0% survival; Ac, 25.3% survival) or solvent (only 20.0%), indicating the effective therapeutic role of the Ac+FLC combination in treating nematodes with C. albicans infections (Fig. 2C). Moreover, the toxicity assay demonstrated that 2.0 μM Acs did not cause significant detrimental effects on the survival of noninfected C. elegans within 5 days (Fig. 2D). These data suggested the potential application of Ac+FLC in treating C. albicans infection *in vivo*.

### Combination of Ac and FLC increased fungal membrane permeability of FLC-resistant C. albicans.

**FIG 3:**
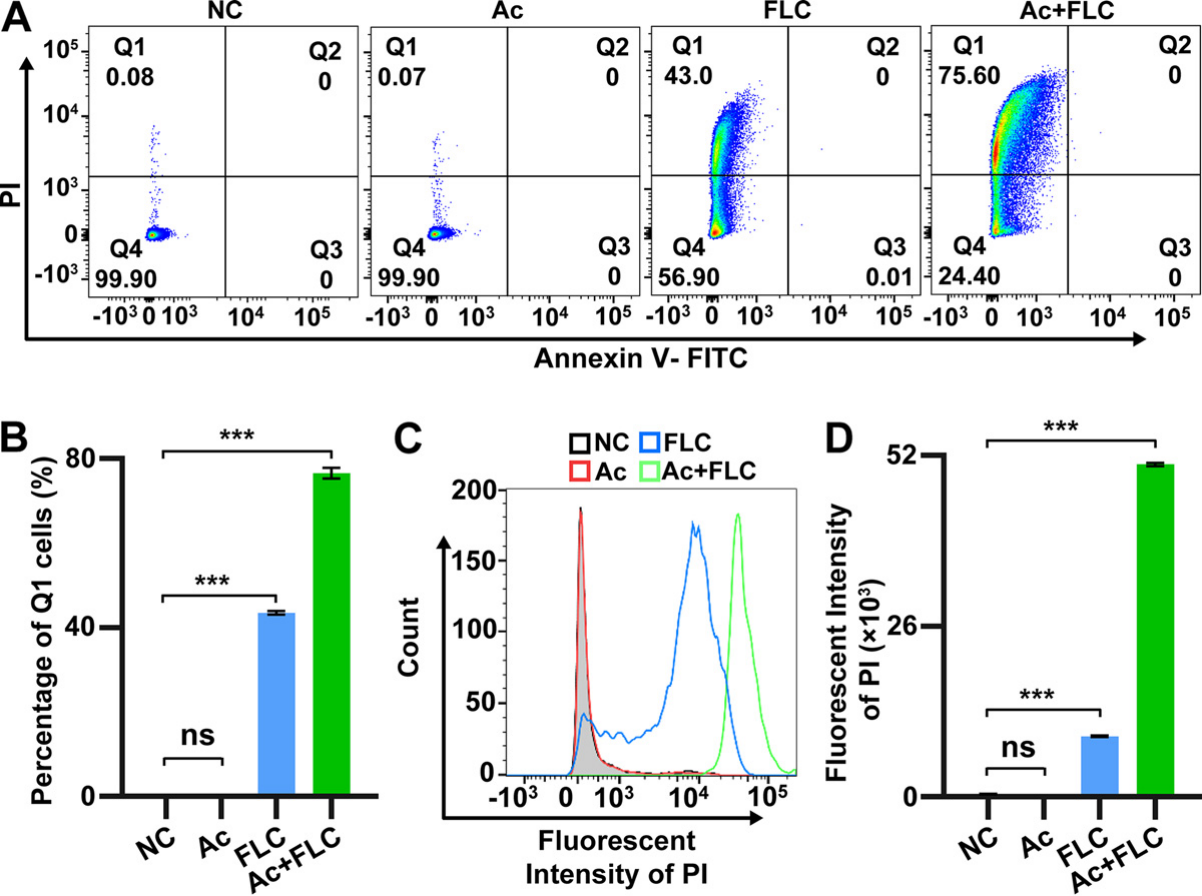
The effect of Ac+FLC on the fungal membrane permeability of FLC-resistant C. albicans. NC, conidia treated with DMSO; Ac, conidia treated with Acs; FLC, conidia treated with FLC; Ac+FLC, conidia treated with Ac+FLC. (A) The annexin V/PI staining analysis of fungal conidia. Q4, living cells, (annexin V-FITC)^−^/PI^−^; Q3, early apoptosis, (annexin V-FITC)^+^/PI^−^; Q2, late apoptosis, (annexin V+FITC)^+^/PI^+^; Q1, (annexin V-FITC)^−^/PI^+^. (B) Quantitative analysis of PI-positive cells in Q1. ***, *P *< 0.001, two-tailed Student's *t* test. Data represent mean ± SD from three independent biological replicates. (C and D) Fluorescence-activated cell sorter (FACS) and quantitative analysis of cell membrane permeability with PI staining. ***, *P *< 0.001, two-tailed Student's *t* test. ns, no significant difference (*P* > 0.05). Data represent mean ± SD from three independent biological replicates.

The effects of the Ac+FLC combination on fungal apoptosis were evaluated by using annexin V/propidium iodide (PI) staining with flow cytometry. Living fungal cells in the Ac group were quite similar to those in the NC group (Q4; 99.9% in NC versus 99.9% in Ac) and so were the apoptotic cells (early apoptosis and late apoptosis, Q2 + Q3) (Fig. 3A and B). Interestingly, the living cells in the Ac+FLC group were extremely decreased (Q4; 24.4% in Ac+FLC versus 99.9% in NC), but there was no significant change in the percentages of apoptotic cells (Fig. 3A and B). Notably, the Ac+FLC group displayed dramatically increased PI-positive cells (Q1) compared with those in the NC or Ac group (75.6% in Ac+FLC versus 0.08% in NC and 0.07% in Ac) (Fig. 3C and D). Meanwhile, we also observed that the PI-positive cells were significantly increased in the FLC group (43.00% in FLC versus 0.08% in NC). FLC has been reported to target Erg11, which is responsible for the biosynthesis of ergosterol that is an important component for fungal cell membrane (12–15). Previous studies suggested that some of the PI-positive cells of FLC-resistant strains were still alive and appeared to have intact membranes, and PI acted as a marker for the fungal membrane permeability because FLC could significantly increase PI in FLC-resistant C. albicans strains without affecting fungal growth and survival (23, 24). We also observed that the PI-positive conidia of the FLC-resistant C. albicans strain were also still alive with intact membrane, consistent with the previous studies (23, 24). This suggested that Ac+FLC could mostly increase the membrane permeability of the FLC-resistant C. albicans strain.

### Accumulation of Acs in the fungal cells were induced by FLC.

**FIG 4:**
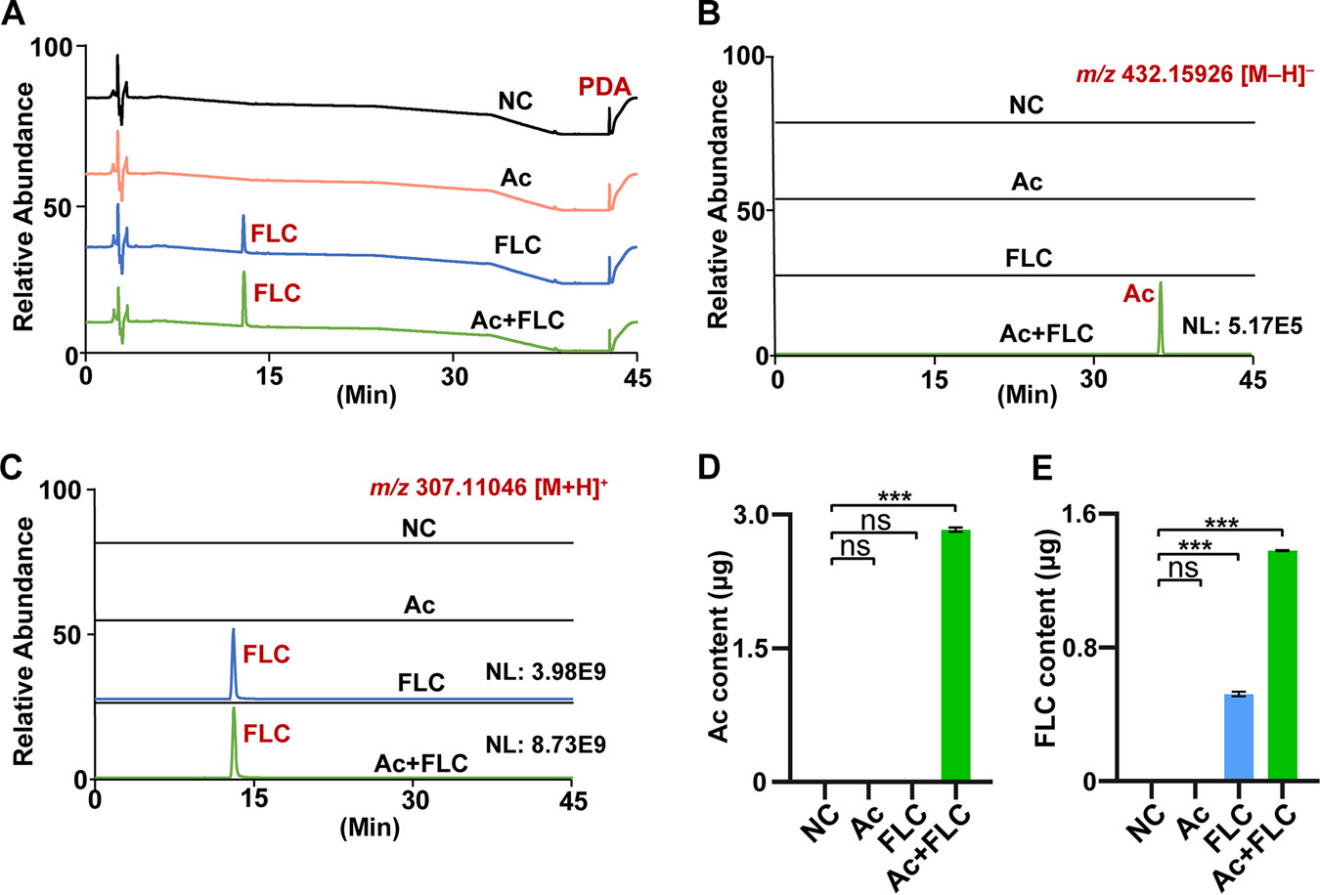
The accumulation of intracellular Acs and FLC induced by Ac+FLC. NC, conidia treated with DMSO; Ac, conidia treated with Acs; FLC, conidia treated with FLC; Ac+FLC, conidia treated with Ac+FLC. (A to C) High-performance liquid chromatography-photodiode array mass spectrometry (HPLC-PDA/MS) analysis of intracellular Ac and FLC; the target peaks for the intracellular FLC (blue) and Ac (red) in PDA (A), [M−H]^−^ (B) and [M+H]^+^ (C). (D and E) The quantitative analysis of the intracellular contents of FLC (blue) and Ac (red). Data represent mean ± SD from three independent biological replicates. ***, *P* value < 0.01, two-tailed Student's *t* test. ns, no significant difference (*P *> 0.05).

Considering the increased cell permeability of FLC-resistant C. albicans in the presence of FLC and Ac+FLC, we assumed that FLC might induce the biological effect of Acs by improving intracellular bioavailability. Using ultraperformance liquid chromatography with diode array detection mass spectrometry (UPLC-DAD/MS) analysis, we evaluated the intracellular levels of Ac and FLC in FLC-resistant C. albicans after treatment with (i) the combination of 2.0 μM Ac and 8 μg/mL FLC, (ii) 2.0 μM Ac, and (iii) 8 μg/mL FLC, respectively, for 24 h at 30°C. Interestingly, no intracellular Acs could be detected in the fungus treated with Ac alone (Fig. 4A to D), which explained why no inhibitory activity for Ac alone toward the FLC-resistant C. albicans was observed. However, 43.7% of Acs used in the Ac+FLC treatment were detected inside the cells of the FLC-resistant C. albicans strain (Fig. 4D), suggesting that FLC could induce the accumulation of Ac in the cells of FLC-resistant C. albicans. At the same time, we found that the intracellular level of FLC in the fungal cells treated with the Ac+FLC combination also increased by 1.65 times compared with those treated with FLC alone (Fig. 4E). However, the above result suggested that the increased intracellular level of FLC could not inhibit the FLC-resistant C. albicans. Thus, we deduced that the intracellular Acs might play a key role in inhibiting FLC-resistant C. albicans from the inside. Overall, metabolic profiles of the FLC-resistant C. albicans strain in four different drug treatments suggested that the presence of FLC could induce the accumulation of Acs in the fungal cells and the presence of Ac could also enhance the intracellular accumulation of FLC.

### Ac+FLC caused abnormal cell walls and mitochondria in C. albicans.

**FIG 5:**
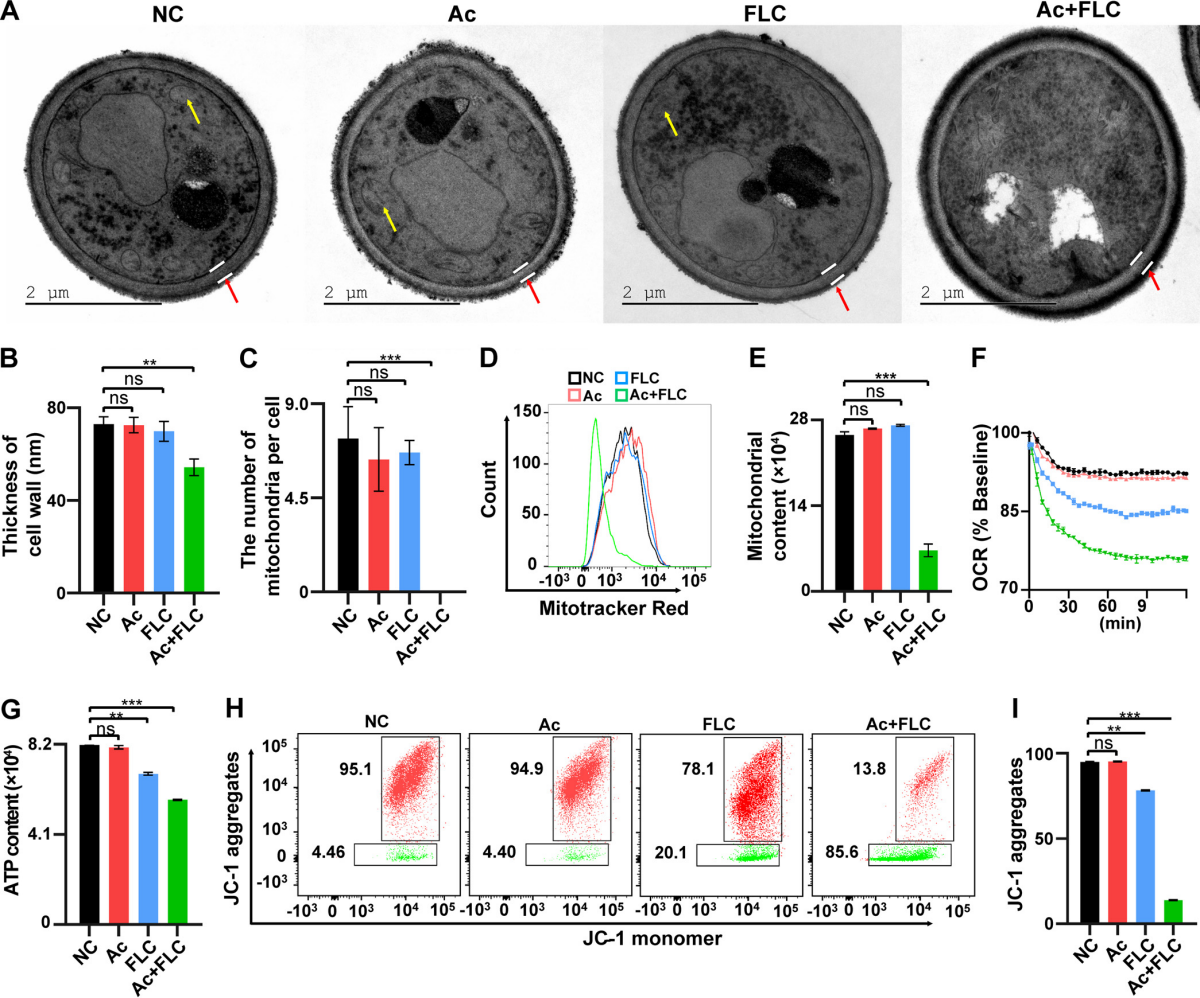
The effects of Ac+FLC on the infrastructures of FLC-resistant C. albicans and mitochondrial functions. NC, conidia treated with DMSO; Ac, conidia treated with Acs; FLC, conidia treated with FLC; Ac+FLC, conidia treated with Ac+FLC. (A) TEM analysis of fungal conidia. The cell membranes, mitochondria, and vacuoles in the Ac+FLC group were distinctly different from those in the NC, Ac, and FLC groups. Mitochondria, yellow arrows; cell wall, red arrows. (B and C) Quantitative analysis of the number of mitochondria (B) in conidia and the thickness of fungal cell walls (C) with TEM analysis (A). (D and E) Representative histograms and quantitative analysis of mitochondrial contents in conidia with MitoTracker red staining. Data represent results from three independent experiments. (F and G) Quantitative analysis of OCR (F) and ATP level (G) in conidia. (H and I) Analysis of mitochondrial membrane potential (MMP) with JC-1 staining. Reduction of MMP prevented the accumulation of JC-1 in the mitochondria, and JC-1 was dispersed throughout the cells, leading to a shift from red (JC-1 aggregates) to green fluorescence (JC-1 monomers). **, *P *< 0.01; ***, *P *< 0.001, two-tailed Student's *t* test (C, E, G) and two-way ANOVA (D); ns, no significant difference (*P* > 0.05). Data represent mean ± SD from at least three independent biological replicates.

Subsequently, we took advantage of methods for ultrathin sectioning to examine the infrastructure of FLC-resistant C. albicans by transmission electron microscopy (TEM) (25). The untreated C. albicans cells displayed smooth and intact cell walls, homogeneous cytoplasm, and intact organelles (Fig. 5A). In contrast, exposure to the combination of 2.0 μM Ac and 8 μg/mL FLC caused uneven plasma density and increased mucus protein protrusions on the cell wall, leading to abnormal cell walls (Fig. 5A). We also analyzed the average thickness of the cell wall of C. albicans in the four groups (Fig. 5A and B). The thickness of the cell wall in the Ac+FLC group (127.23 ± 2.97 nm) was significantly smaller than those in the other three groups (NC, 37.68 ± 1.77 nm; Ac, 98.44 ± 13.78 nm; FLC, 39.91 ± 2.83 nm) (*P* < 0.001) (Fig. 5B).

Importantly, intact mitochondria were dramatically decreased in the Ac+FLC group compared with those in the other three groups (Fig. 5A). Detailed analysis revealed that the number of normal mitochondria in the combination group (0) was much lower than that in the other three groups (NC, 8 ± 1/cell; Ac, 5 ± 1/cell; FLC, 7 ± 1/cell) (*P* < 0.01) (Fig. 5C). Moreover, the characteristic membranes for the key large organelles, such as normal large vacuoles in the fungal cells in the other three groups could hardly be distinguished in the Ac+FLC group (Fig. 5A). These results indicated that the combination of Ac+FLC could cause abnormal formation of the cell wall and intracellular organelles, including distinct mitochondria and vacuoles.

### Ac+FLC induced mitochondrial dysfunction in C. albicans.

The hallmarks of mitochondrial demise included reduced mitochondrial content and ATP levels and loss of mitochondrial membrane potential (MMP) (26). We, thus, evaluated mitochondria content, MMP, and ATP level, as well as oxygen consumption rate (OCR), in the four treatments of solvent, Ac, FLC, and Ac+FLC. Flow cytometry analysis of mitochondrial staining with 100 nM MitoTracker red revealed that the mitochondrial content in FLC-resistant C. albicans treated with the combination of Ac+FLC (673.3) was dramatically lower than that of either drug alone (FLC, 2,718; Ac, 2,659) or solvent (NC, 2,557) (Fig. 5D and E). The mitochondrial content in the combination of Ac+FLC dropped by 3.04 times compared with that of FLC alone (Fig. 5D and E). Both the OCR and ATP levels in the combination of Ac+FLC (75.99% for OCR; 5,671 for ATP) were significantly lower than in either drug alone (FLC, 85.06% and Ac, 91.52% for OCR; FLC, 6,859 and Ac, 8,063 for ATP) or solvent (NC, 92.17% for OCR; fluorescence intensity, 8,159 for ATP) (Fig. 5F and G). For the MMP assay, fungal conidia were stained with the cationic lipid fluorescent dye JC-1. The conidia treated with the combination of Ac+FLC displayed dramatically decreased MMP (JC-1 monomer, 13.80%) compared with that of the other three groups (NC, 94.93%; Ac, 95.17%; FLC, 78.23%) (*P* < 0.001) (Fig. 5H and I).

### Transcriptional link of Ac+FLC to increased membrane transports but decreased fungal virulence.

**FIG 6:**
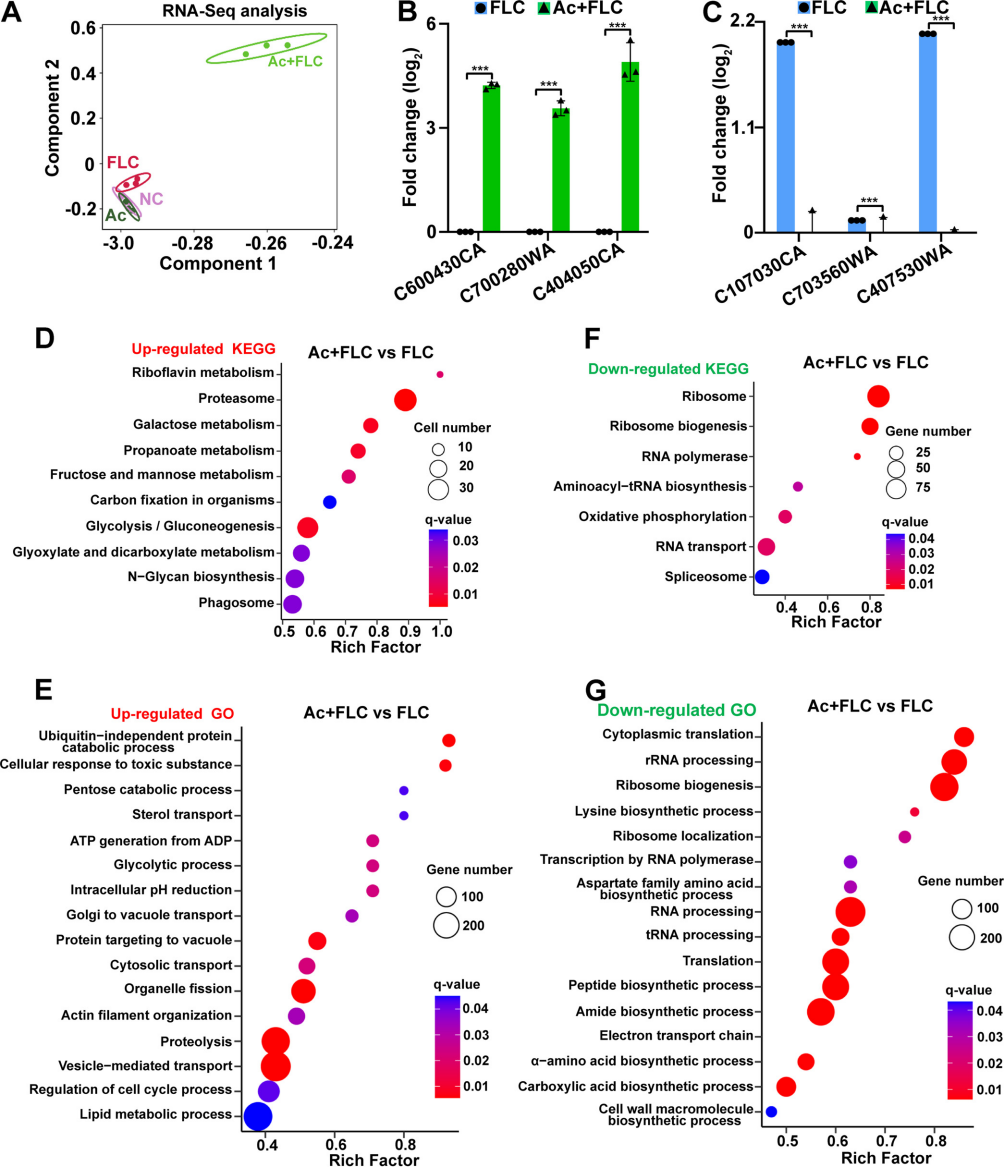
Transcriptomic and qRT-PCR analysis of the effect of Ac+FLC on FLC-resistant C. albicans. (A) Principal-component analysis (PCA) of the DEGs in FLC-resistant C. albicans treated with NC, Ac, FLC, and Ac+FLC, respectively. (B and C) The top 3 upregulated (B) and downregulated (C) DEGs in Ac+FLC versus FLC samples. Data are presented as mean ± SD of technical triplicates. ***, *P* value < 0.01, two-tailed Student's *t* test. (D and E) KEGG pathway analysis and GO classification of all of the upregulated DEGs in Ac+FLC versus FLC groups. (F and G) KEGG pathway analysis and GO classification of all of the downregulated DEGs in Ac+FLC versus FLC groups. The *x* axis represents the gene ratio, which refers to the ratio of DEG numbers annotated in the pathway term to all gene numbers annotated in the pathway term. The circle size indicates the number of DEGs that are associated with each significant pathway. The circle color indicates the significant level with the adjusted *P* value < 0.05, one-sided version of Fisher’s exact test.

To further explore the potential antifungal mechanism involved in the Ac+FLC combination, we compared the transcriptomic profiles of the fungal strains in four different treatments. Principal-component analysis (PCA) analysis of the samples from four treatments showed that NC, Ac, and FLC samples distributed in adjacent locations, while the Ac+FLC samples were independent of the other three groups (Fig. 6A), indicating that the Ac+FLC treatment stood in sharp contrast to the other three treatments. In Ac+FLC versus FLC, 1,736 differentially expressed genes (DEGs) were significantly upregulated and 1,745 DEGs were significantly downregulated (DESeq, *P* ≤ 0.05) (see Table S2 in the supplemental material). Among them, the most upregulated genes in Ac+FLC versus FLC were *C600430CA*, *C700280WA*, and *C404050CA* (Fig. 6B). Importantly, the top 1 upregulated gene *C600430CA* remained totally silent in either FLC or Ac alone because no transcriptional levels of this gene could be detected representative transcriptional levels (RTL, 0), which is in sharp contrast to the high transcriptional level (RTL, 501) in the Ac+FLC treatment. The top 2 and 3 upregulated genes displayed dramatically increased transcriptional levels in Ac+FLC by 67.2 and 35.3 times than those in FLC only, respectively (Fig. 6C). These top 3 upregulated genes were putatively assigned as the MHD domain, Hgt12p (hexose transporter), and cell wall protein RHD3, respectively. Interestingly, the MHD domain was involved in the membrane-tubulation activity associated with transmembrane cargo proteins. Moreover, among the top 20 upregulated genes in Ac+FLC versus those in FLC (see Table S3 and Fig. S2 in the supplemental material), except 5 unknown DEGs, 11 DEGs were involved in membrane transports and cell wall, which is consistent with the dramatically increased cell membrane permeability and the accumulation of Acs in the above results.

Interestingly, the top 3 downregulated genes were *C107030CA*, *C403570WA*, and *C703560WA* (Fig. 6C), assigned as important virulence factor RBT4p, hyphal wall adhesion protein 1 (Hwp1p), and virulence factor CaO19.6688, respectively. All three genes were mainly involved in fungal pathogenesis (27–29).

### Riboflavin metabolism and proteasomes were mostly upregulated in the Ac+FLC treatment.

KEGG enrichment analysis of all of the upregulated DEGs in Ac+FLC versus FLC indicated that riboflavin metabolism and proteasomes were the two most upregulated (Fig. 6D). Riboflavin (also known as vitamin B_2_) is an important component of the cofactors flavin adenine dinucleotide (FAD) and flavin mononucleotide (FMN), which play pivotal roles in mitochondrial electron transport chain, β-oxidation of fatty acids, redox homeostasis, and apoptosis (30). A previous study indicated that the disorder of riboflavin metabolism was associated with mitochondrial dysfunction (30). This was consistent with our results from the TEM and transcriptomic analyses. Moreover, GO analysis of all of the upregulated DEGs in Ac+FLC versus FLC revealed that ubiquitin-independent protein catabolic process and cellular response to toxic substance were mostly enriched (Fig. 6E). The enriched ubiquitin-independent protein catabolic process was consistent with the upregulated proteasomes that were a part of a major mechanism by which cells regulated the concentration of particular proteins and degrade misfolded and damaged proteins. The highly upregulated autophagosomes in Ac+FLC (Fig. 6D) were also consistent with previous studies showing that phagosomes contribute to the removal and degradation of cellular garbage (31), most likely due to the accumulation of abnormal proteins.

A previous study suggested that riboflavin metabolism also plays pivotal roles in protein folding (30). Combining the upregulated transport processes, including Golgi to vacuole transport, protein targeting to vacuole, cytosolic transport, and vesicle-mediated transport, we deduced that abnormal proteins might be accumulated in the fungus treated with the Ac+FLC combination, thus leading to the most upregulated ubiquitin-independent protein degrading process. This might also explain why ribosome and ribosome biogenesis were the most downregulated KEGG pathways in the Ac+FLC treatment (Fig. 6F) because ribosome is an important cell organelle composed of RNA and protein that are responsible for protein synthesis. This was also consistent with the top three downregulated processes, cytoplasmic translation, rRNA processing, and ribosome biogenesis in GO analysis of the Ac+FLC versus FLC (Fig. 6G).

**FIG 7:**
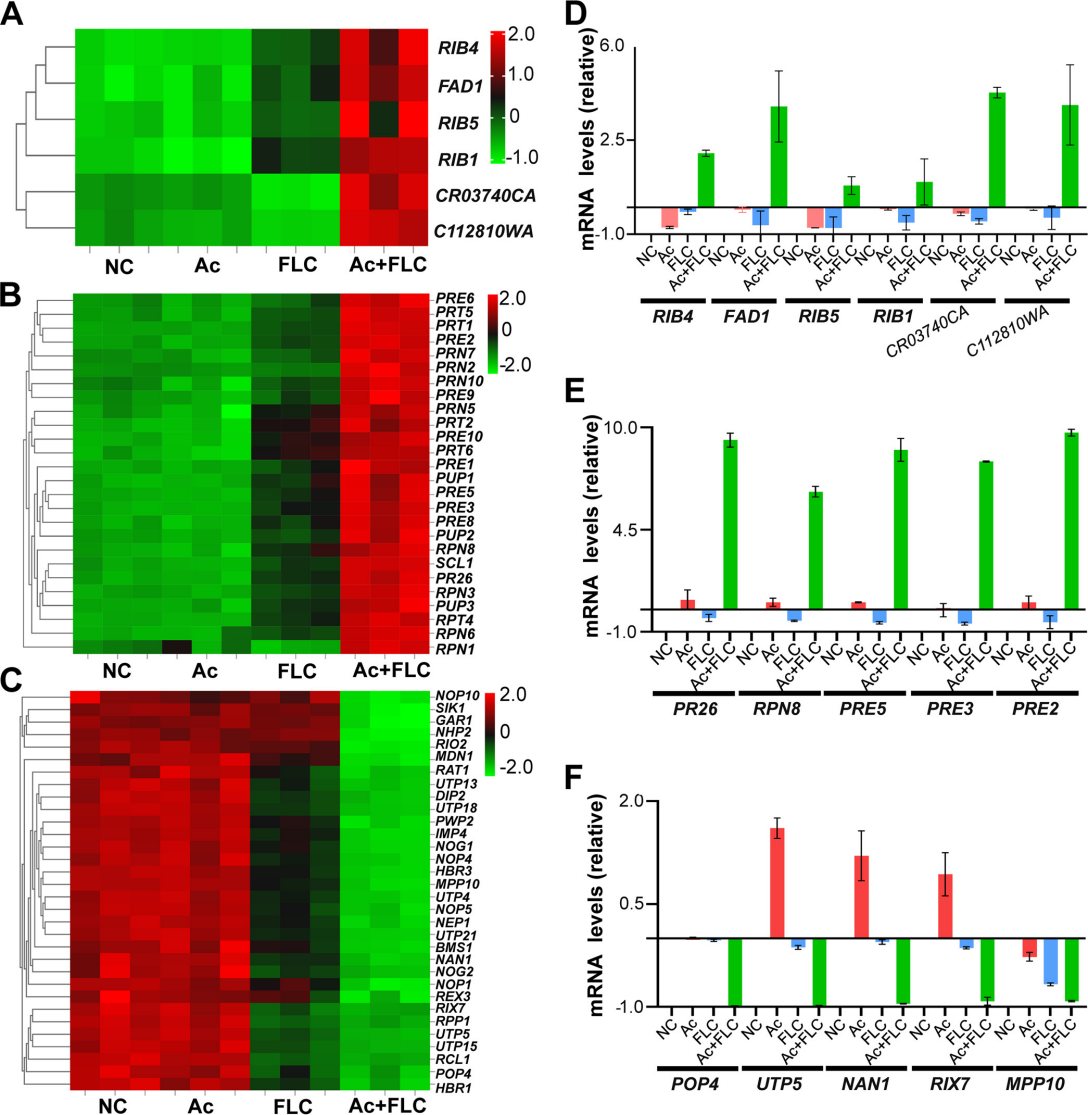
Riboflavin metabolism and proteasomes were upregulated pathways induced by the Ac+FLC treatment. NC, conidia treated with DMSO; Ac, conidia treated with Acs; FLC, conidia treated with FLC; Ac+FLC, conidia treated with Ac+FLC. (A to C) Heat map analysis of the DEGs involved in riboflavin metabolism (A), ribosome (B), and proteasomes (C). Gene expression patterns are in log_10_ scale. Red boxes, upregulated clusters; blue boxes, downregulated clusters. (D to F) qRT-PCR analysis of the transcriptional levels of the key genes involved in riboflavin metabolism (D), ribosome (E), and proteasomes (F).

Further heat map analysis of all of the DEGs involved in riboflavin metabolism, proteasomes, and ribosome revealed that the Ac+FLC combination treatment stood in sharp contrast to the other three treatments (Fig. 7A to C). Detailed reverse transcription-quantitative PCR (qRT-PCR) analysis of the key genes in all three pathways (Fig. 7D to F) confirmed all of the transcriptomic data. The six key genes (*RIB4*, *FAD1*, *RIB5*, *RIB1*, *CR03740CA*, *C112810WA*) involved in riboflavin metabolism in fungal conidia treated with Ac+FLC were upregulated 0.8 to 4.3 times compared with those treated with FLC alone (Fig. 7D). The 5 key genes (*POP4*, *UTP5*, *NAN1*, *RIX7*, *MPP10*) involved in ribosome pathways in the fungus treated with Ac+FLC dropped by 0.9 to 1.0 times compared with those in fungus treated with FLC alone (Fig. 7E).

### Ac+FLC inhibited protein biosynthesis and promoted protein degradation and autophagy.

**FIG 8:**
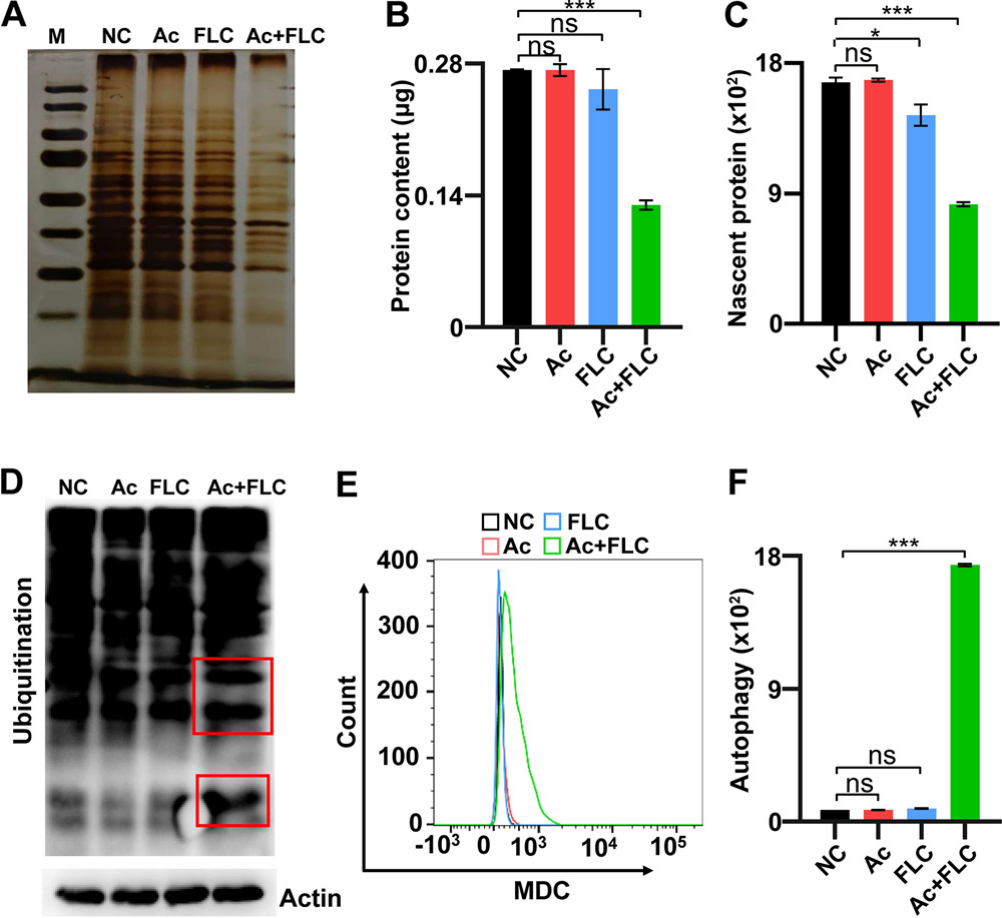
The effects of Ac+FLC on total protein, ubiquitylation, and autophagy levels in FLC-resistant C. albicans. NC, conidia treated with DMSO; Ac, conidia treated with Acs; FLC, conidia treated with FLC; Ac+FLC, conidia treated with Ac+FLC. (A) Silver stain analysis of the total protein levels in fungal conidia. (B and C) Quantitative analysis of total protein (B) and nascent protein levels (C) in conidia. (D) Western blotting of the ubiquitylation levels of the total protein in conidia. Red boxes refer to the upregulated ubiquitylation levels. (E and F) FACS and quantitative analysis of the autophagy levels with MDC staining. *, *P *< 0.05; ***, *P *< 0.001, two-tailed Student's *t* test. ns, no significant difference (*P* > 0.05). Data represent mean ± SD from at least three independent biological replicates.

We evaluated the protein levels in NC-, Ac-, FLC-, and Ac+FLC-treated samples with the same number of cells. Silver staining protein assay showed a dramatic decrease in the total protein level in the Ac+FLC group compared with that in the other three samples (Fig. 8A). Further quantitative protein analysis displayed that the total protein level in the FLC-resistant C. albicans treated with the combination of Ac+FLC (0.13 μg/10^8^ cells) was dramatically lower than that of either drug alone (FLC, 0.25 μg/10^8^ cells; Ac, 0.27 μg/10^8^ cells) or solvent (NC, 0.27 μg/10^8^ cells) (Fig. 8B). We further evaluated the nascent protein levels by using flow cytometer analysis with methionine analog l-homopropargylglycine (HPG) (32, 33). The nascent protein level in FLC-resistant C. albicans treated with the combination of Ac+FLC (734.1) was dramatically lower than either drug alone (FLC, 1281.0; Ac, 1496.3) or solvent (NC, 1481.3) (Fig. 8C). Interestingly, we also found that the ubiquitination level of most proteins in the Ac+FLC group was also significantly increased compared with that of the other groups normalized to internal control β-actin levels (Fig. 8D). These results indicate that the combination of Ac+FLC could significantly reduce protein levels via inhibiting protein biosynthesis and ribosome biogenesis and promoting protein degradation, which is totally consistent with the transcriptional results. Flow cytometer analysis of autofluorescent monodansylcadaverin (MDC) as a marker of autophagosomes revealed that the fluorescence intensity in Ac+FLC (1,738.0) was much higher than that in the other three groups (NC, 79.8; Ac, 79.2; FLC, 86.7) (*P* < 0.001) (Fig. 8E and F), suggesting that Ac+FLC could largely increase autophagosomes. These results were totally consistent with most upregulated proteasome and most downregulated ribosome and ribosome biogenesis in the combination of Ac+FLC.

### Ac+FLC combination increased reactive oxygen species/superoxide anion and lipids.

**FIG 9:**
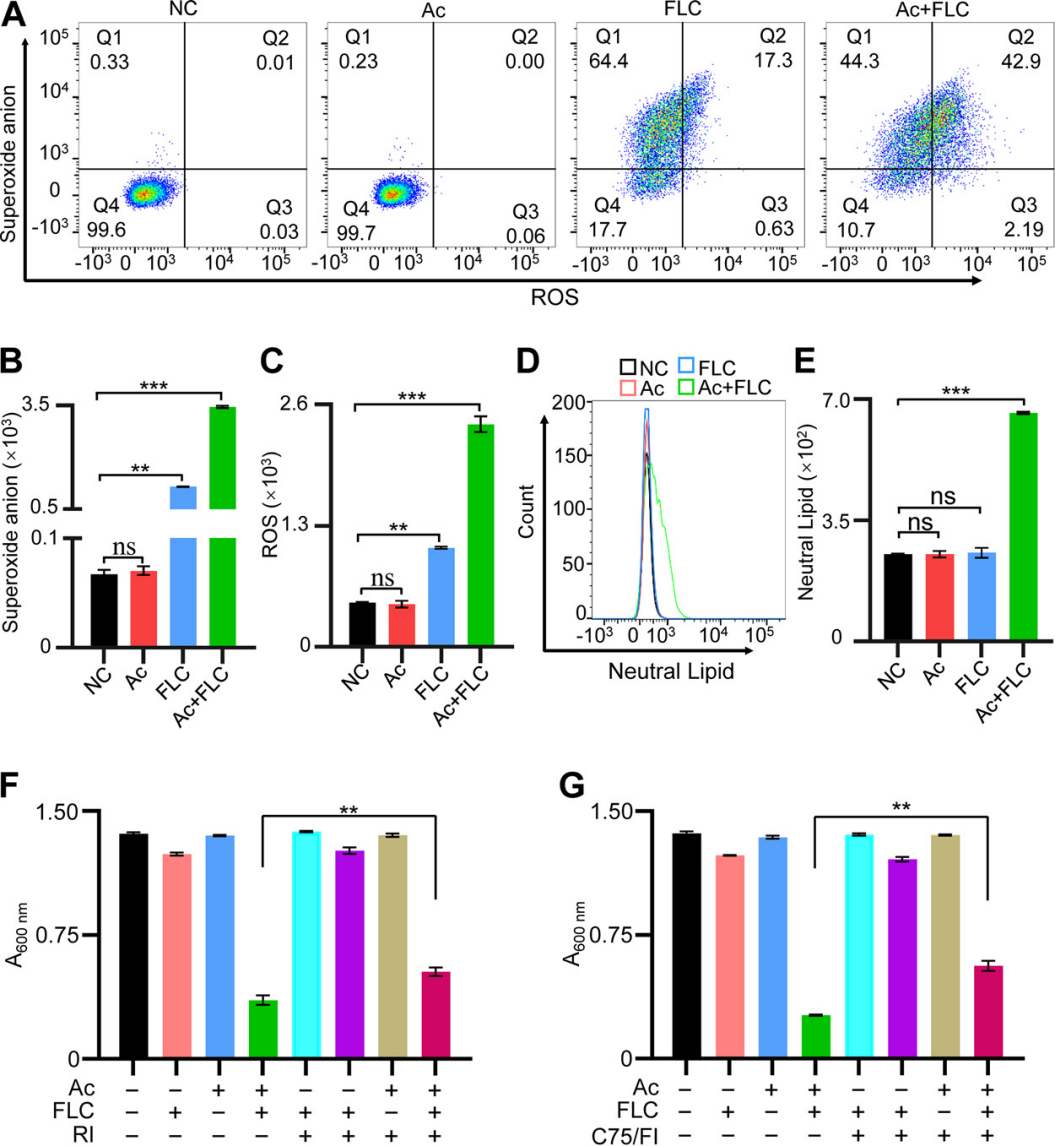
The effect of Ac+FLC on the cellular ROS, superoxide anion, and neutral lipid levels in FLC-resistant C. albicans. NC, conidia treated with DMSO; Ac, conidia treated with Acs; FLC, conidia treated with FLC; Ac+FLC, conidia treated with Ac+FLC. (A to C) Representative and quantitative analysis of intracellular superoxide anion (B) and ROS (C) levels in conidia. Q4, negative cell population; Q3, ROS-positive cell population; Q2, both ROS and superoxide anion-positive cell population; Q1, superoxide anion-positive cell population. (D and E) Representative histograms and quantitative analysis of neutral lipid levels in conidia. Data represent results from three independent experiments with 3 samples. (F and G) Analysis of the fungal conidia treated with ROS inhibitor *N*-acetyl-l-cysteine at the concentration of 5 mM (F) and FASN inhibitor C75 at the concentration of 50 μM (G). **, *P *< 0.01; ***, *P* < 0.001, two-tailed Student's *t* test. ns, no significant difference (*P* > 0.05). Data represent mean ± SD from at least three independent biological replicates.

Because both riboflavin metabolism and mitochondrial function played vital roles in redox homeostasis and lipid metabolism, ROS/superoxide anion detection assay and neutral lipid level analysis were performed in the FLC-resistant C. albicans strain with four different treatments (Fig. 9A to G). FLC-resistant C. albicans treated with the combination of Ac+FLC displayed significantly higher ROS and superoxide anion levels (2,389 and 3,461) than those of samples treated with either drug alone (FLC, 1,063 and 1,162; Ac, 458 and 70) or solvent (NC, 474 and 67) (Fig. 9A to C). Moreover, the neutral lipid level in FLC-resistant C. albicans treated with the combination of Ac+FLC (660.3) was significantly higher than that in FLC-resistant C. albicans treated with either drug alone (FLC, 256.0; Ac, 251.7) or solvent (NC, 251.6) (Fig. 9D and E). Furthermore, ROS inhibitor (RI) and fatty acid synthase inhibitor (FI) were applied to detect whether excess accumulation of ROS and lipid mediated by Ac+FLC affected the fungal growth. As expected, RI and FI significantly rescued the fungal survival in the Ac+FLC group but displayed few effects on the other groups (Fig. 9F and G). These results suggested that the combination of Ac+FLC caused the excess accumulation of ROS and lipids in the FLC-resistant C. albicans.

## DISCUSSION

This study addresses the mechanism of Ac, a potent anticancer fluorescent dye-like complex xanthene, that greatly “restored” the sensitivity of intractable human pathogen FLC-resistant Candida albicans to FLC. We indicated that Acs could synergize with FLC against FLC-resistant C. albicans. The Ac+FLC combination dramatically increased the survival of 293T human cells and the nematode Caenorhabditis elegans infected with FLC-resistant C. albicans. Importantly, synergizing with FLC at 8 μg/mL, Acs at less than 2 μM could cause fungal cell damage and death, suggesting that Acs should be a new class of potential antifungal compounds (34).

We found that FLC could induce fungal membrane permeability to Ac and the accumulation of Acs in fungal cells. Meanwhile, Acs could also enhance the intracellular FLC levels. However, we also observed that FLC at much higher concentrations could not inhibit FLC-resistant C. albicans. Thus, we proposed that the trace intracellular Acs in the fungal conidia (about 0.86 μM) induced by FLC might play a critical role in inhibiting FLC-resistant C. albicans.

The combination of Ac+FLC may cause thinner fungal cell walls with many mucus protein protrusions. What is more, using TEM, we could not discern the intact mitochondria in the fungal cells treated with the Ac+FLC combination. A series of mitochondrial function assays suggested the largely reduced mitochondria contents and ATP levels, oxygen consumption rate, and loss of mitochondrial membrane potential, indicating mitochondrial dysfunctions. This was consistent with loss of intact mitochondria in the fungal cells treated with the Ac+FLC combination.

Transcriptomic and qRT-PCR analyses revealed that FLC-resistant C. albicans treated with the Ac+FLC combination had a distinct character that differentiated it from that treated with the other three treatments, including Ac alone, FLC alone, and solvent alone. The 3 most upregulated genes with the Ac+FLC treatment versus the FLC treatment were involved in the membrane-tubulation activity associated with membrane transports, consistent with the dramatically increased cell membrane permeability and the accumulation of Acs in the fungal cells treated with the Ac+FLC combination. Interestingly, the 3 most downregulated genes in Ac+FLC versus FLC belonged to three important virulence factors that were associated with hyphal wall adhesion proteins and mainly involved in fungal pathogenesis. This may also explain why the Ac+FLC treatment could obviously increase the survival rates of 293T human cells and nematodes infected with FLC-resistant C. albicans by inhibiting the fungal hyphal wall pathogenic adhesion proteins.

We found that the Ac+FLC treatment could also cause the upregulation of riboflavin metabolism and proteasome pathways in the FLC-resistant C. albicans. Riboflavin metabolism has been commonly found to play pivotal roles in the mitochondrial electron transport chain, β-oxidation of fatty acids, redox homeostasis, citric acid cycle, branched-chain amino acid catabolism, chromatin remodeling, DNA repair, and protein folding (30). Riboflavin has been used as a potential therapeutic agent in the “mitochondrial cocktail,” particularly in complex I- and II-related primary mitochondrial disease to ameliorate oxidative stress (35). The upregulated riboflavin metabolism in the Ac+FLC group versus the FLC group was consistent with mitochondrial dysfunction and the loss of intact mitochondria in the Ac+FLC treatment. This may also explain why proteasomes were the top 2 upregulated pathways in the Ac+FLC treatment group. This is likely because of false protein folding induced by riboflavin metabolism and mitochondrial dysfunction. Furthermore, the quantitative analysis of protein levels, including nascent proteins, revealed that the Ac+FLC treatment inhibited the nascent protein biosynthesis and decreased protein levels in FLC-resistant C. albicans. Combining the fact that the autophagy level was highly increased in the Ac+FLC treatment group, the accumulation of abnormal proteins likely due to false folding may lead to the upregulated autophagy pathway responsible for removing and degrading the false proteins and the most downregulated pathways for protein biosynthesis. Further results show that large increases in the ROS/superoxide anion and lipid levels in the Ac+FLC treatment were consistent with riboflavin metabolism disorder and mitochondrial dysfunction.

A recent study suggested that the riboflavin pathway served as one potentially attractive metabolic pathway in antibacterial discovery. In this strategy, ribocil, a highly selective chemical modulator of bacterial riboflavin riboswitches, repressing riboswitch-mediated *ribB* gene expression and inhibiting bacterial cell growth, was screened out of an internal library of 57,000 synthetic small molecules (36). A number of drugs have been reported to sensitize *Candida* cells to FLC mainly by affecting their calcineurin pathway, Hsp90 activity and other Hsp mediated stress response pathway, and the target of rapamycin (TOR) pathway (37–39). Among them, several adjuvant drugs, when used together with FLC, were reported to significantly increase intracellular FLC levels and augment the effect of FLC on the fungal cells, for example, artificially synthesized azoffluxin that could enhance FLC activity through inhibition of efflux pump *Cdr1* (21). Another well-known antibacterial agent colistin, which targeted lipopolysaccharides within the membranes of Gram-negative bacteria, could also bind to fungal membrane lipids and permeabilize fungal cells to antifungal azoles (24). However, until now, investigations on the effects of any compounds on the riboflavin pathway in fungi are still lacking. We believe that this is the first discovery of fluorescent dye-like xanthenes via targeting the riboflavin metabolism and proteasomes pathways and then inhibiting intractable fluconazole-resistant Candida albicans. The unprecedented structures of arthrocolins with their impressive biological activity will also provide a new incentive for exploring unexpected biosynthesis of seminaturally occurring metabolites from microbes.

In summary, our study suggested that Ac did restore the sensitivity of FLC-resistant C. albicans to FLC. The synergism of Ac with FLC occurs through a series of steps. In the presence of FLC, Acs could enter the fungal cells, suggesting that FLC could induce fungal permeability to Acs. The intracellular Acs could induce the disorder of riboflavin metabolism and proteasomes and mitochondrial dysfunction. This increased the ROS and autophagy levels, leading to fungal cell death. Importantly, the antifungal activity of Ac+FLC occurs in a dose-dependent manner of Acs, such that Acs can be used with FLC at low concentrations, which might be developed into potential clinical drugs for fungal infection.

## MATERIALS AND METHODS

### Agents.

Acs A to C were isolated from Escherichia coli (E_BL21_) fermentation according to the previously described methods (16). Fluconazole was purchased from Sigma (catalog no. F8929). *N*-acetyl-l-cysteine was purchased from Abcam (catalog no. 139476), and fatty acid synthase (FASN) inhibitor was purchased from Sigma (catalog no. C5490). All of the compounds were dissolved in 1mL dimethyl sulfoxide (DMSO).

### Fungal strain cultivation.

The FLC-resistant Candida albicans kzs01 (40) and FLC-sensitive C. albicans SC5314 (41) were provided by the Dermatological Department of Kunming Medical University and stored at the Laboratory for Conservation and Utilization of Bio-Resources & Key Laboratory for Microbial Resources of the Ministry of Education, Yunnan University, Kunming. C. albicans kzs01 and SC5314 were suspended in yeast peptone dextrose medium (YPD) (1% yeast extract, 2% peptone, 2% glucose), yeast peptone dextrose plates (YPD plates) (1% yeast extract, 2% peptone, 2% glucose, 2% agar), or RPMI 1640 medium (Gibco; catalog no. 11875176) at 30°C. Fungal growth was quantified by measuring optical density at 600 nm (OD_600_) and corrected for medium background. Fungal conidia were collected by washing the 3-day cultures on one 9-cm YPD plate with 1 mL of phosphate buffer solution (PBS) (Sigma; catalog no. P5244). The raw conidial solution was centrifuged at 1,000 × *g* for 10 min in 1.5-mL tubes, and the supernatant was removed. The conidia were resuspended in 1 mL of YPD medium to prepare a stock conidial solution.

### Chemical susceptibility assays.

Compound potency was assessed alone by dose-response assays or in combination with another compound by dose-response matrixes in 96-well plates (Corning). Two hundred microliters of 1 × 10^5^/mL fungal conidia was added to each well with 200 μL of YPD medium in a 96-well plate. Meanwhile, tested compounds (NC, 2 μL of DMSO; Ac, 1 μL of arthrocolin solution and 1 μL of DMSO; FLC, 1 μL of FLC solution and 1 μL of DMSO; Ac+FLC, 1 μL of arthrocolin solution and 1 μL of FLC solution) were added to each well and then incubated at 30°C with shaking overnight. Arthrocolin solutions were set at concentrations of 0.5, 1.0, 2.0, 4.0, and 8.0 μM. The concentrations of FLC solution were set at 50, 100, 200, 400, and 800 μg/mL for the FLC-sensitive C. albicans strain and at 800, 1,600, 3,200, 6,400, and 12,800 μg/mL for the FLC-resistant C. albicans strain. Fungal growth was quantified by measuring the OD_600_ value on an Envision plate reader and corrected for medium background. Fungal growth was normalized to untreated controls and plotted as a bar chart using GraphPad Prism (version 9.3.0). Dose-response matrixes fractional inhibitory concentration index at 90% growth inhibition (FICI_90_) was calculated using the following formula: (MIC_Drug A Combo_/MIC_Drug A Alone_ + MIC_Drug B Combo_/MIC_Drug B Alone_). All strains were assessed in biological duplicate experiments with technical duplicates.

### Coculture bioassay of 293T human cell line and FLC-resistant C. albicans.

Human cell line 293T was purchased from the Kunming Cell Bank of the Chinese Academy of Science. Dulbecco modified Eagle medium (DMEM) (Gibco, Carlsbad, USA; catalog no. C11995500CP) specially for 293T culture was supplemented with 10% fetal bovine serum (BI, Kibbutz Beit Haemek, Israel; catalog no. 04-001-1ACS) and 1% penicillin/streptomycin (Gibco; catalog no. C0222). 293T cells were cultured in DMEM in a 5% CO_2_ humidified environment. To assess the ability of Ac+FLC to rescue mammalian cell growth in coculture experiments, 5 × 10^5^ 293T cells in 100 μL of DMEM were added to each well of a 96-well plate and incubated at 37°C in 5% CO_2_ overnight. Then, the old DMEM was replaced with 100 μL of fresh DMEM. Meanwhile, 1 μL of 2.5 × 10^6^/mL C. albicans kzs01 conidia suspended in 1 μL of PBS was added to each well with 293T cells in DMEM. Then, test compounds (NC, 2 μL of DMSO; Ac, 1 μL of 100 μM arthrocolins and 1 μL of DMSO; FLC, 1 μL of 800 μg/mL FLC and 1 μL of DMSO; Ac+FLC, 1 μL of 100 μM arthrocolins and 1 μL of 800 μg/mL FLC) were added into each well with the cocultures of 293T and FLC-resistant C. albicans. The 96 plates with the cocultures were incubated at 37°C in 5% CO_2_. After 48 h, the cell survival rates of 293T were detected by Steady-Glo luciferase assay reagent (Promega; catalog no. E2520) according to the manufacturer’s instructions (42), and the fluorescence intensity was measured on a microplate reader. C. albicans and 293T were stained with a periodic acid-Schiff (PAS) staining kit (Solarbio; catalog no. G1280) and hematoxylin (Sigma; catalog no. H3136) according to the manufacturer’s instructions. The plates were air dried and then visualized with an Olympus microscope.

### C. elegans-C. albicans infection model.

The *in vivo* antifungal activities of compounds were evaluated using the C. elegans-C. albicans kzs01 infection model as previously reported (43). Age-synchronous populations of Caenorhabditis elegans nematodes were collected with 10 mL M9 buffer (3 g/L KH_2_PO_4_, 6 g/L Na_2_HPO_4_, 5 g/L NaCl, and 1 mM MgSO_4_) and were washed three times with 5 mL of PBS in 50-mL tubes (Corning; catalog no. 430304). Briefly, 1 × 10^4^ FLC-resistant C. albicans conidia were added to one 9-cm plate containing 10 mL of brain heart infusion (BHI) solid medium (Solarbio; catalog no. LA0360) (BHI, 38.5 g; agar [Solarbio; catalog no. A8190], 12.5 g; distilled water, 1 L). Meanwhile, 50 nematodes were added to the center of the 9-cm plate with BHI plates containing C. albicans and then incubated for 2 h at 25°C. Then, the 50 C. elegans worms were transferred into a 50-mL tube and washed with PBS 3 times. The 50 C. elegans worms were then transferred into a well of a new 6-well plate with 3 mL 20% (g/mL) BHI solution (20 g of BHI dissolved in 99 mL of PBS plus 1 mL of 4.5 mg/mL kanamycin) supplemented with tested compounds (NC, 2 μL of DMSO; Ac, 1 μL of 200 μM arthrocolins and 1 μL of DMSO; FLC, 1 μL of 800 μg/mL FLC and 1 μL of DMSO; Ac+FLC, 1 μL of 200 μM arthrocolins and 1 μL of 800 μg/mL FLC). The worms were assumed to be dead if no response was detected after prodding with a platinum wire. The survival rates of infected worms were monitored with an Olympus microscope daily. The statistical significance (*P* value) of differences between the groups was analyzed by the Kaplan-Meier survival method.

### HPLC-DAD/MS analysis for intracellular FLC and Ac.

FLC-resistant C. albicans was subcultured from overnight cultures at a starting OD_600_ of 0.4 in 5 mL of YPD supplemented with tested compounds (NC, 100 μL of DMSO; Ac, 50 μL of 100 μM arthrocolin solution and 50 μL of DMSO; FLC, 50 μL of 800 μg/mL FLC solution and 50 μL of DMSO; Ac+FLC, 50 μL of 100 μM arthrocolin solution and 50 μL of 800 μg/mL FLC solution) and then incubated at 30°C with shaking overnight. Fungal cultures were then transferred to falcon tubes and centrifuged at 3,000 × *g* for 5 min at 4°C. The medium was removed, and fungal conidia were washed with 5 mL of cold PBS three times with centrifugation of 2,000 × *g* for 5 min in between. Fungal conidia were resuspended in 1 mL cold PBS, flash frozen in liquid nitrogen, and stored at −80°C overnight. The fungal conidia solutions were thawed on ice on the next day. Twenty-five microliters of 6 N NaOH (Oklabs; catalog no. 10019762; 12 g dissolved in 50 mL of double-distilled water [ddH_2_O]) was added to the fungal conidia solution and then vortexed for 15 s. Next, 500 μL of 10 mM sodium phosphate (pH 6.0) was added to each sample followed by vortexing for 15 s. Compounds were extracted with 5 mL of CH_2_Cl_2_ and vortexed for 5 min, followed by centrifugation for 10 min at 4,000 × *g* at 4°C. The organic phase was collected and dried. Before high-performance liquid chromatography mass spectrometry (HPLC-MS) analysis, samples were dissolved in 500 μL of CH_3_OH (20). The resuspended fungal conidia extracts (10 μL) were separated on an Agilent Zorbax eclipse XDB-C_18_ column (5 μm, 4.6 × 250 mm) using the Acquity UPLC I-Class coupled to an electrospray Q Exactive Focus mass spectrometer equipped with an electrospray ionization (ESI) source. Chromatography followed a gradient method (A, water + 0.1% [vol/vol] HCOOH; B, C_2_H_3_N; 0 to 40 min, 10% B to 95% B at 1.0 μL min^−1^). FLC and Ac were detected using authentic samples and mass ion peaks at 307.110 [M+H]^+^ and 432.159 [M−H]^−^. TargetLynx (Waters) was used for peak finding, smoothing, and area calculations. All samples were run in biological duplicate and technical triplicate, and a representative replicate was plotted in GraphPad Prism.

### Fungal conidia of FLC-resistant strain treated with tested compounds.

About 5 × 10^5^ fungal conidia in 5 mL of YPD medium were added in each well of a 6-well plate. Meanwhile, tested compounds (NC, 100 μL of DMSO; Ac, 50 μL of 100 μM arthrocolin solution and 50 μL of DMSO; FLC, 50 μL of 800 μg/mL FLC solution and 50 μL of DMSO; Ac+FLC, 50 μL of 100 μM arthrocolin solution and 50 μL of 800 μg/mL FLC solution) were added in each well and then incubated at 30°C with agitation overnight. Fungal conidia were pelleted at 1,000 × *g* for 10 min in 15-mL tubes (Corning; catalog no. 430052), and the media was removed. The fungal conidia were transferred to a 1.5-mL tube (Axygen; catalog no. MCT-150-C) and washed with PBS three times. The fungal conidia were used for a series of assays and analysis including cell permeability, apoptosis, transmission electron microscopy, autophagy, mitochondrial membrane potential (MMP), oxygen consumption rate (OCR), reactive oxygen species (ROS), mitochondrial level, ATP, nascent protein detection, protein level, and quantitative real-time PCR (RT-qPCR).

### Cell permeability assay with propidium iodide staining.

The fungal conidia were resuspended in 1 mL PBS supplemented with 1 μL fluorometric reagent for 30 min at room temperature in the dark. The conidia were resuspended in 1 mL of PBS. Then, 1 μL of 5 mg/mL propidium iodide (PI) solution (25 mg PI [Sigma; catalog no. P4170] dissolved in 5 mL PBS) was added into fungal conidia and incubated in the dark for 30 min. Subsequently, the fungal conidia were collected and washed as above. The conidia were then analyzed with flow cytometry, and the data were analyzed using FlowJo X software (23).

### Apoptosis assay.

Apoptosis was detected using an annexin V-FITC/PI detection kit (BD Pharmingen; catalog no. 556547) as previously reported (44). The fungal conidia were suspended in 1 mL of binding buffer solution (25 μL of annexin-FITC and 25 μL of PI in 950 μL of binding buffer in the kit) and incubated for 20 min at room temperature. Subsequently, the fungal conidia were collected and washed as above. The fungal conidia were then analyzed by flow cytometry, and the data were analyzed using FlowJo X software.

### Transmission electron microscopy.

The fungal conidia were resuspended in 1 mL of glutaraldehyde fixative (Sigma; catalog no. G7651) for 4 h at 4°C. The fungal conidia were centrifuged, and the supernatants were removed. The fungal conidia were washed with calcium carbonate buffer solution three times. Then, the fungal conidia were treated with 200 μL 1% (g/mL) agarose solution (Tsingke; catalog no. TSJ001; 0.1 g, dissolved in 10 mL of ddH_2_O at 55°C) and cooled at room temperature. Then, the samples were postfixed with 1% OsO_4_ (Sigma; catalog no. 05500) for 2 h at room temperature in the darkness. After the OsO_4_ was removed, the samples were rinsed in PBS 3 times, each for 15 min, followed by serial ethanol dehydration (30%, 50%, 70%, 80%, 90%, 95%, and 100%) and acetone (100%) transition for 5 min each, embedding in Epon 812 resin (Shell Chemicals; catalog no. GS02660) with polymerization for 48 h at 60°C. Thin slices (60 to 80 nm thick) were obtained by using a diamond cutter (Diatome, Ultra 45°) on a Leica superslicer (Leica UC7). The samples were then stained with a uranium acetate-saturated alcohol solution (8 min) and lead citrate (8 min) (24). The samples were analyzed and photographed using TEM (Hitachi; HT7800/HT7700). The thickness of C. albicans cell walls were quantitatively analyzed using Image-Pro Plus image analysis software version 7.0.1 (Media Cybernetics Inc.).

### Autophagy assay.

The fungal conidia were suspended in 1 mL of PBS in a 1.5-mL tube, and 50 μM dansylcadaverine (Sigma-Aldrich; 30432) was used for staining at 37°C for 15 min. The fungal conidia were washed with PBS three times and resuspended in 1 mL of PBS followed by analysis with flow cytometry. The data were analyzed using FlowJo X software.

### Intracellular neutral lipid content detection.

Lipid assay kit (Abcam; catalog no. ab242307) was used for neutral lipid detection assay. The fungal conidia were suspended in 1 mL PBS supplemented with 1 μL of lipid fluorometric reagent for 30 min at room temperature in the darkness. Then, the stained cells were collected and washed with cold PBS three times. The fungal conidia were resuspended in 1 mL of PBS and fluorescence intensities were measured at excitation (Ex)/emission (Em) = 490/585 nm by flow cytometry, and the data were analyzed using FlowJo VX software.

### Mitochondrial membrane potential assay.

The MMP in C. albicans was analyzed by JC-1 staining using a previously reported method (45). The fungal conidia were suspended in 1 mL of PBS supplemented with 1 μL of 2 μM JC-1 (MCE; catalog no. HY-K0601) at 30°C for 1 h in the darkness. Then, the stained cells were collected and washed with cold PBS three times. The fungal conidia were resuspended in 1 mL of PBS and fluorescence intensities. The fluorescence densities of the JC-1 aggregates (red) and monomer (green) were recorded by a flow cytometer. A total of 10,000 events were collected for data analysis. The data were analyzed using FlowJo VX software.

### Oxygen consumption rate assay.

Mitochondrial stress test complete assay kit (Abcam; catalog no. ab197243) was applied to detect the OCR according to the manufacturer’s instructions. About 100 μL of 1 × 10^6^/mL fungal conidia (1 × 10^5^ cells/well) was seeded in 96-well plates. Then, 8 μL of reconstituted extracellular O_2_ consumption reagent and 100 μL (or 2 drops) of prewarmed high-sensitivity mineral oil (37°C) were added to each well. The extracellular O_2_ consumption signal at 1.5-min intervals for ~90 to ~120 min at Ex/Em = 380/650 nm was recorded with a microplate reader. The extracellular O_2_ probe is quenched by O_2_ through molecular collision. Thus, the amount of fluorescence signal is inversely proportional to the amount of extracellular O_2_ in the samples. After baseline oxygenation was established, the OCRs were calculated from the changes in the fluorescence signal over time.

### Reactive oxygen species assay.

ROS/superoxide detection assay kit (Abcam; catalog no. ab139476) was used to monitor production of reactive oxygen species (ROS). The fungal conidia were stained with 500 μL of ROS/superoxide detection solution for 1 h at 30°C in the dark. Then, the stained cells were collected and washed with cold PBS three times. The fungal conidia were resuspended in 1 mL PBS, fluorescence intensities were analyzed by flow cytometry, and the data were analyzed using FlowJo VX software.

### Mitochondrial contents assay.

Mitochondrial contents (26) were measured by MitoTracker red CMXRos (Invitrogen; catalog no. M7514). The fungal conidia were suspended in 1 mL of PBS supplemented with 1 μL of 1 mM MitoTracker red CMXRos for 1 h at 30°C in the dark. Then, the fungal conidia were centrifuged and washed with PBS three times. The fungal conidia were resuspended in PBS, followed with flow cytometry analysis at Ex/Em = 490/516 nm. The data were analyzed using FlowJo VX software.

### ATP level.

ATP content assay kit (Solarbio; catalog no. BC0300) was used to evaluate intracellular ATP levels according to the manufacturer’s instructions. One milliliter of extract solution was added into a 1.5-mL tube containing 1 × 10^8^ fungal conidia. Then, the fungal conidia were ground into powder on ice and ultrasonically disrupted at 200 W for 2 s. The fungal conidia solution was centrifuged at 1,000 × *g* for 10 min at 4°C, and the supernatant was transferred into a 1.5-mL tube. Next, 500 μL of CHCl_3_ were added into the tube and mixed. Then, the mixture was centrifuged at 1,000 × *g* for 3 min at 4°C, and the supernatant was collected for detection. Creatine kinase catalyzes creatine and ATP to produce phosphocreatine. In order to detect ATP level, the content of phosphocreatine was determined by colorimetric method of phosphomolybdic acid. The absorbance at 700 nm was measured by a microplate reader.

### Nascent protein assay.

Click-iT HPG Alexa Fluor protein synthesis assay kits (Thermo Fisher; catalog no. C10429) were used to evaluate nascent protein level. About 1 × 10^6^ fungal conidia were added into a 1.5-mL tube with 1 mL of methionine-free medium for 30 min prior to the addition of 100 μL of 50 μM methionine analog l-homopropargylglycine (HPG) for 90 min. After incubation, HPG was removed, and fungal conidia were washed with PBS. Then, 1 mL of 3.7% formaldehyde (Solarbio; catalog no. P1110) was added into the tube containing the fungal conidia and incubated for 15 min at room temperature. After incubation, 3.7% formaldehyde was removed and fungal conidia were washed twice with 1 mL of 3% bovine serum albumin (BSA) (Solarbio; catalog no. A8020; 3 g, dissolved in 100 mL of PBS). After washing, 1 mL of 0.5% Triton X-100 in PBS (5 μL of Triton X-100 [Solarbio; catalog no. T8200] dissolved in 955 μL of PBS) was added into the tube and incubated for 20 min at room temperature. After incubation, the supernatant was removed, and fungal conidia were washed with 1 mL of 3% BSA twice. Then, 1 mL of Click-iT reaction buffer (21.5 mL of Click-iT HPG reaction buffer, 1.0 mL of copper sulfate, 62.5 μL of Alexa Fluor azide, and 2.5 mL of Click-iT HPG buffer additive) was added into the tube and incubated for 30 min at room temperature in the dark. The reaction supernatant was removed, and the sediments were washed with 1 mL of Click-iT reaction rinse buffer once. After washing, 1 mL of 1× HCS NuclearMask blue stain solution was added and incubated for 30 min at room temperature in the dark. After the Blue Stain solution was removed, the sediments were washed with PBS twice and then resuspended in 1 mL PBS, followed with flow cytometry at Ex/Em = 590/617 nm (29). The data were analyzed using FlowJo VX software.

### Ubiquitination assay.

Total proteins of 1 × 10^8^ fungal conidia were extracted using 300 μL of radioimmunoprecipitation assay (RIPA) buffer (Beyotime; catalog no. P0013J) on ice. The supernatants were collected and boiled at 100°C for 7 min. Then, the total protein extracts were separated by electrophoresis and transferred to polyvinylidene fluoride membranes. The following primary antibodies were used: anti-ubiquitin (Cell Signaling; catalog no. 3936) and anti-α-tubulin (ProteinTech; catalog no. 11224-1-AP).

### Protein level analysis.

Analysis of protein levels with silver staining were carried out according to a previously described method (46). Total proteins of 1 × 10^8^ fungal conidia were extracted with 300 μL of RIPA buffer (Beyotime; catalog no. P0013J) on ice. The supernatants were collected and boiled at 100°C for 10 min. Then, the total protein extracts were separated by electrophoresis. Proteins were fixed in 40% ethanol/5% acetic acid (vol/vol) (40 mL of ethanol [Sigma; catalog no. 1012768] and 5 mL of acetic acid [Sigma; catalog no. A6283] dissolved in ddH_2_O to 100 mL) for 30 min and then in 10% ethanol/5% acetic acid (vol/vol) (10 mL of ethanol and 5 mL of acetic acid dissolved in ddH_2_O to 100 mL) for 30 min, followed with oxidization for 5 min in a Bio-Rad oxidizer (1 mL of Bio-Rad oxidizer in the kit dissolved in 9 mL of ddH_2_O). Then, the proteins were washed with ddH_2_O for 15 min and incubated with silver reagent (1 mL of silver reagent dissolved in 9 mL of ddH_2_O) for 20 min. Next, the stained gels were washed with ddH_2_O for 1 min and developed with 10 mL of developer (Bio-Rad; developer, 32 g in 1 L ddH_2_O) for 10 min, followed with being stopped in 5% acetic acid (vol/vol) (5 mL of acetic acid dissolved in ddH_2_O to 100 mL). The gels were visualized with an Olympus microscope.

### RNA extraction, sequencing, and differential gene expression analysis.

Total RNAs were extracted from about 1 × 10^8^ fungal conidia per sample of FLC-resistant strains C. albicans between the control group and different drug-regimen groups using the RNeasy Plus kit (Qiagen; catalog no. 74136) following the manufacturer’s instructions. RNA integrity and quantitation were assessed using a NanoDrop spectrophotometer (Thermo Scientific) and a Bioanalyzer 2100 (Agilent Technologies). Complementary DNA (cDNA) library preparation was performed at Beijing Genomics Institution (BGI) (Beijing, China). All qualified samples were sequenced on a BGISEQ-500 sequencing platform generating ~6 GB 150-bp paired-end reads per sample. The transcriptome sequencing (RNA-seq) reads were first processed with fastp (v 0.23.2) to trim both adapter sequences and low-quality base calls (Phred quality score, <25) using default settings (47). All filtered reads were mapped to the FLC-sensitive C. albicans SC5314 reference genome (version A22) using HISAT2 (version 2.2.1) with specific parameters “–dta –very-sensitive” (48). Raw read counts were obtained with featureCounts. Differential gene expression analysis was performed with the R package DESeq2 (49). Wald test was used to identify genes that are differentially expressed between every two sample groups. Differentially expressed genes (DEGs) were identified based on adjusted *P* value (BH) of <0.05 and |log2FoldChange (FC)| of >0 as selection criteria as described in a previous study (50). The clusterProfiler (version 4.2.2) package was used to evaluate enrichment of the GO and KEGG categories in the sets of upregulated and downregulated genes separately (51). The categories with adjusted *P* value (BH) below 0.05 were considered significantly enriched.

### Quantitative real-time-PCR.

The RNAs of 1 × 10^8^ fungal conidia were extracted using the AxyPrep Multisource Total RNA miniprep kit (Axygen; catalog no. AP-MN-MS-RNA-250). cDNA synthesis was performed using the PrimeScript RT reagent kit with genomic DNA (gDNA) eraser (TakaRa; catalog no. RR047Q). Quantitative real-time PCR was performed using Fast SYBR green master mix (Tsingke; catalog no. TSE201) and the Bio-Rad CFX-96 real time system with the following cycling conditions: 95°C for 3 min and then 95°C for 10 s and 60°C for 30 s, for 40 cycles. The melt curve was completed with the following cycle conditions: 95°C for 10 s and 65°C for 10 s with an increase of 0.5°C per cycle up to 95°C. All of the primers used in this study are listed in Table S4 in the supplemental material.

### Statistical analysis.

The data were expressed as mean ± standard deviation (SD). All experiments were performed at least three times. Significant differences were analyzed by two-way analysis of variance (ANOVA) or two-tailed Student's *t* test using GraphPad Prism 9.3.0. A *P* value of <0.05 was considered statistically significant.

### Data availability.

All of the data and methods necessary to reproduce this study are included in the manuscript and supplemental material. The raw data from the transcriptomic experiment have been deposited in the SRA database of NCBI under SRA accession no. PRJNA874924.
