# Supplementary material for: Arthrocolins Synergizing with Fluconazole Inhibit Fluconazole-Resistant Candida albicans by Increasing Riboflavin Metabolism and Causing Mitochondrial Dysfunction and Autophagy
Source: Microbiol Spectr. 2023 Feb 27;11(2):e04051-22. doi: 10.1128/spectrum.04051-22 (PMC10101122; doi:10.1128/spectrum.04051-22)
Supplement: Supplemental file 1 — Supplemental material. Download spectrum.04051-22-s0001.pdf, PDF file, 0.6 MB [file spectrum.04051-22-s0001.pdf]

SUPPLEMENTAL MATERIAL

Legends:

Figure S1. (A–B) Chemical susceptibility assays for FLC–sensitive *C. albicans* were performed in YPD (A–B) and RPMI 1640 media (C–D) supplemented with (A and C) FLC (0 to 8 μg/mL), (B and D) AcB or AcB+FLC, in 2–fold dilution. FLC inhibited the growth of FLC–sensitive *C. albicans* in a concentration–dependent manner. Error bars represent means ± S.D. (E–F) Chemical susceptibility assays for FLC–resistant *C. albicans* were performed in RPMI 1640 medium supplemented with (E) FLC (0 to 8 μg/mL), (F) AcB or AcB+FLC. FLC+AcB inhibited the growth of FLC–resistant *C. albicans* in a concentration–dependent manner in a concentration–dependent manner of Acs. Error bars represent means ± S.D. The growth of FLC–resistant *C. albicans* in each well is presented in line chart based on the OD_{600} value at 24 h relative to the negative control without drug treatments (NC).

Figure S2. Quantitative analysis of the top 20 up-regulated DEGs (A) and down-regulated DEGs (B) in Ac+FLC vs. FLC samples.

TABLE S1. (A) MIC_{90} and $FICI_{90}$ values were calculated based on chemical susceptibility assays with FLC–resistant *C. albicans*. The fungal

growth in each well was presented based on the OD_{600} values relative to NC at 24 h interval in YPD medium supplemented with (0 to 1024 μg/mL) and Acs (0 to 8 μM). The $FICI_{90}$ values for Acs A–C (0.5−8 μM) in the presence of FLC (8 μg/mL) were 0.03, 0.02, and 0.03, respectively. (B) The $FICI_{90}$ value for AcB (0.5−8 μM) with FLC (1 μg/mL) was 0.25, with $FICI_{90}$ value < 0.5 indicating a synergistic interaction.

TABLE S2. Differentially expressed genes (DEGs) in Ac+FLC treatment vs. FLC treatment.

TABLE S3. Top 20 up-regulated and down-regulated genes in Ac+FLC treatment vs. in FLC treatment.

TABLE S4. The list for all the primes used in this study.

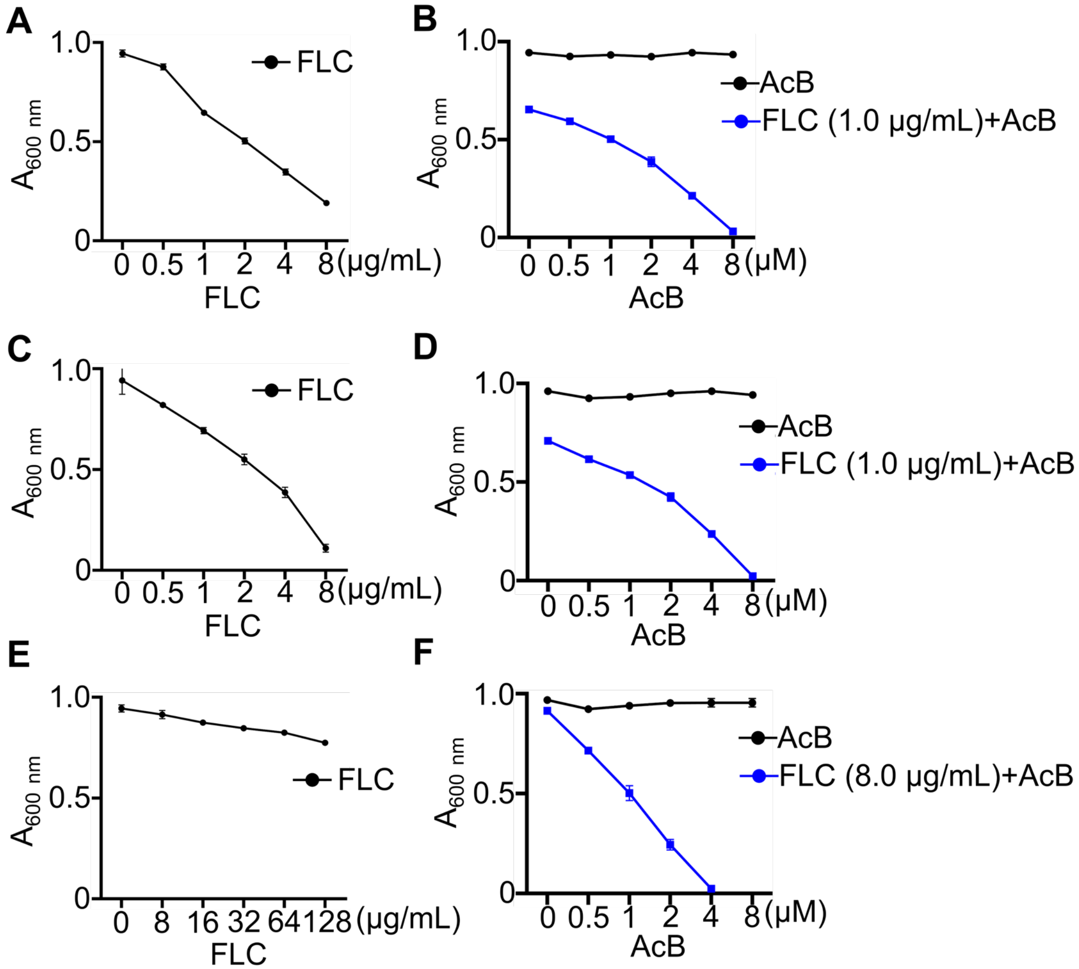

Figure S1 (A−B) Chemical susceptibility assays for FLC−sensitive *C. albicans* were performed in YPD (A−B) and RPMI 1640 media (C−D)

supplemented with (A and C) FLC (0 to 8 μg/mL), (B and D) AcB or AcB+FLC, in 2−fold dilution. FLC inhibited the growth of FLC−sensitive *C. albicans* in a concentration−dependent manner. Error bars represent means ± S.D. (E–F) Chemical susceptibility assays for FLC−resistant *C. albicans* were performed in RPMI 1640 medium supplemented with (E) FLC (0 to 8 μg/mL), (F) AcB or AcB+FLC. FLC+AcB inhibited the growth of FLC−resistant *C. albicans* in a concentration−dependent manner in a concentration−dependent manner of Acs. Error bars represent means ± S.D. The growth of FLC−resistant *C. albicans* in each well is presented in line chart based on the OD_{600} value at 24 h relative to the negative control without drug treatments (NC).

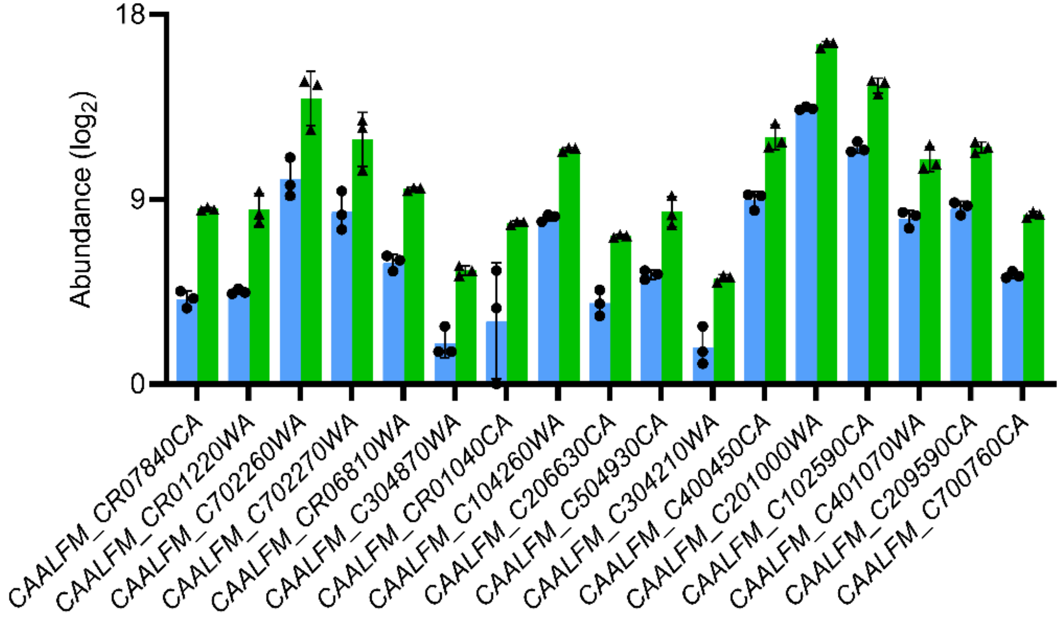

Abundance (log2)
18
9
0
CAALFM_CR07840CA
CAALFM_CR01220WA
CAALFM_C702260WA
CAALFM_C702270WA
CAALFM_CR06810WA
CAALFM_C304870WA
CAALFM_CR01040CA
CAALFM_C104260WA
CAALFM_C206630CA
CAALFM_C504930CA
CAALFM_C304210WA
CAALFM_C400450CA
CAALFM_C201000WA
CAALFM_C102590CA
CAALFM_C401070WA
CAALFM_C209590CA
CAALFM_C700760CA

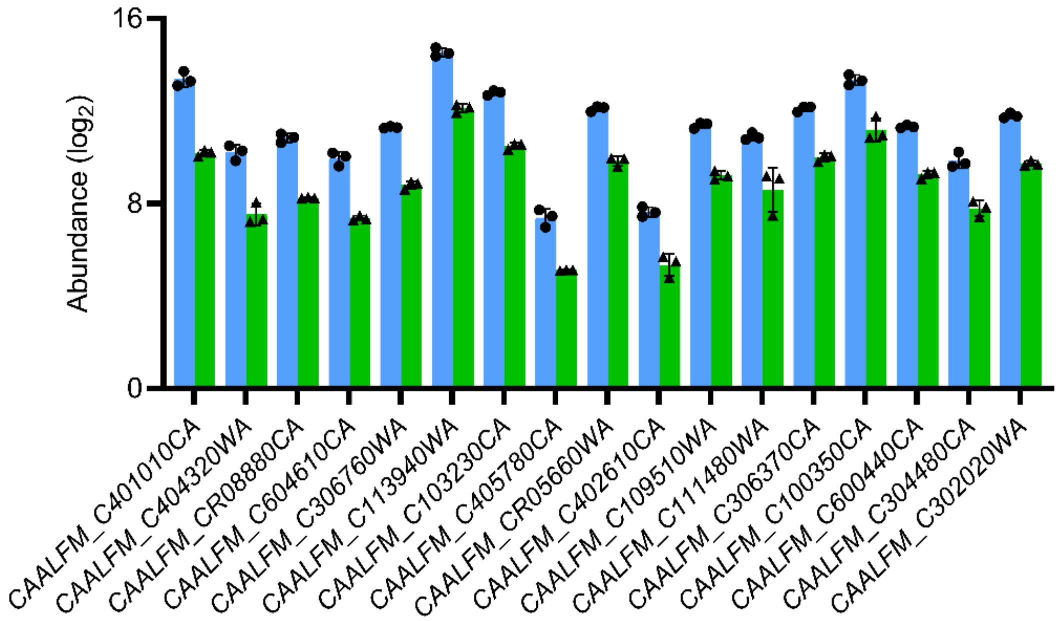

Figure S2 Quantitative analysis of the top 20 up-regulated DEGs (A) and down-regulated DEGs (B) in Ac+FLC vs. FLC samples.

TABLE S1. (A) MIC_{90} and $FICI_{90}$ values were calculated based on chemical susceptibility assays with FLC–resistant *C. albicans*. The fungal growth in each well was presented based on the OD_{600} values relative to NC at 24 h interval in YPD medium supplemented with (0 to 1024 µg/mL) and Acs (0 to 8 µM). The $FICI_{90}$ values for Acs A–C (0.5–8 µM) in the presence of FLC (8 µg/mL) were 0.03, 0.02, and 0.03, respectively. (B) The $FICI_{90}$ value for AcB (0.5–8 µM) with FLC (1 µg/mL) was 0.25, with $FICI_{90}$ value < 0.5 indicating a synergistic interaction.

<table>
<tr><td colspan="6">Towards FLC-resistant C. albicans strain</td></tr>
<tr><td>MIC_{90}</td><td>MIC_{90}</td><td colspan="2">MIC_{90}</td><td>$FICI_{90}$</td><td>Interaction</td></tr>
<tr><td>Ac A</td><td>FLC</td><td>Ac A</td><td>FLC</td><td rowspan="2">0.03</td><td rowspan="2">Syn</td></tr>
<tr><td>>1000 µM</td><td>>1024 µg/mL</td><td>2.04 µM</td><td>8.0 µg/mL</td></tr>
<tr><td>Ac B</td><td>FLC</td><td>Ac B</td><td>FLC</td><td rowspan="2">0.02</td><td rowspan="2">Syn</td></tr>
<tr><td>>1000 µM</td><td>>1024 µg/mL</td><td>1.41 µM</td><td>8.0 µg/mL</td></tr>
<tr><td>Ac C</td><td>FLC</td><td>Ac C</td><td>FLC</td><td rowspan="2">0.03</td><td rowspan="2">Syn</td></tr>
<tr><td>>1000 µM</td><td>>1024 µg/mL</td><td>2.19 µM</td><td>8.0 µg/mL</td></tr>
<tr><td colspan="6">Towards FLC-sensitive C. albicans strain</td></tr>
<tr><td>MIC_{90}</td><td>MIC_{90}</td><td colspan="2">MIC_{90}</td><td>$FICI_{90}$</td><td>Interaction</td></tr>
<tr><td>Ac B</td><td>FLC</td><td>Ac B</td><td>FLC</td><td rowspan="2">0.25</td><td rowspan="2">Syn</td></tr>
<tr><td>>1000 µM</td><td>4.0 µg/mL</td><td>4.0 µM</td><td>1.0 µg/mL</td></tr>
</table>

TABLE S3. Top 20 up-regulated and down-regulated genes in Ac+FLC treatment vs. in FLC treatment.

Up-regulated															
Locus Tag	EntrezID	FLC1	FLC2	FLC3	FLC+AC1	FLC+AC2	FLC+AC3	FLC(mean)	FLC+AC(mean)	Fold Change	Pvalue	Padj	Sig	Description (Uniprot)	Description (NCBI)
CAALFM_C600430CA	3646089	0	0	0	444	552	508	0	501	#DIV/0!	0.0000	0.0000	Up	MHD domain-containing protein	uncharacterized protein CAALFM_C600430CA
CAALFM_C700280WA	3638048	71	19	94	4075	629	7766	61	4157	67.15	0.0000	0.0000	Up	Hgt12p	Hgt12p
CAALFM_C404050CA	3641212	42	88	133	2892	3815	2871	88	3193	35.28	0.0000	0.0000	Up	Cell wall protein RHD3 (LDG family protein 7) (Predicted GPI-anchored protein 29) (Repressed during hyphae development protein 3)	Rhd3p
CAALFM_CR07840CA	3647346	23	18	13	389	355	361	18	368	19.44	0.0000	0.0000	Up	Uncharacterized protein	uncharacterized protein CAALFM_CR07840CA
CAALFM_CR01220WA	3640406	21	25	22	307	232	668	23	402	16.48	0.0000	0.0000	Up	MFS domain-containing protein	uncharacterized protein CAALFM_CR01220WA
CAALFM_C702260WA	3637002	574	822	2092	24501	5371	27375	1163	19082	15.41	0.0000	0.0000	Up	Uncharacterized protein	uncharacterized protein CAALFM_C702260WA

CAALFM_ C702270WA	3637 001	18 4	30 2	67 2	7375	1363	5686	386	4808	11.46	0.0000	0.0001	Up	Ldg3p	Ldg3p
CAALFM_ CR06810W A	3647 326	45	65	76	748	681	755	62	728	10.74	0.0000	0.0000	Up	Histone H3.1/H3.2	histone
CAALFM_ C304870WA	3635 226	3	3	7	54	46	39	4	46	10.50	0.0000	0.0000	Up	Uncharacterized protein	uncharacterized protein CAALFM_C304870WA
CAALFM_ CR01040CA	3640 418	0	13	46	239	236	216	20	230	10.50	0.0177	0.0310	Up	Actin-related protein 2/3 complex subunit 4	Arc19p
CAALFM_ C104260WA	3644 707	24 0	28 6	30 1	2586	2907	2863	276	2785	9.09	0.0000	0.0000	Up	Histone H3.1/H3.2	Hht21p
CAALFM_ C206630CA	3051 5141	15	10	24	141	155	148	16	148	8.25	0.0000	0.0000	Up	Uncharacterized protein	uncharacterized protein CAALFM_C206630CA
CAALFM_ C504930CA	3636 420	41	34	46	301	215	582	40	366	8.15	0.0000	0.0000	Up	Maltose permease	maltose permease
CAALFM_ C304210WA	3635 185	2	7	3	39	37	31	4	36	8.00	0.0000	0.0000	Up	Uncharacterized protein	uncharacterized protein CAALFM_C304210WA
CAALFM_ C400450CA	3645 009	35 0	59 1	57 7	2987	6629	3527	506	4381	7.66	0.0000	0.0000	Up	GPI-anchored hemophore PGA10 (GPI-anchored protein 10) (Repressed by TUP1 protein 51) (Repressed by TUP1 protein 8)	Pga10p

CAALFM_C201000WA	3638876	10525	11674	10853	101650	99694	84523	11017	95289	7.65	0.0000	0.0000	Up	Hgt7p	Hgt7p
CAALFM_C102590CA	3636803	2560	2689	3587	26637	17723	28082	2945	24147	7.20	0.0000	0.0000	Up	Pyridoxal 5'-phosphate synthase (glutamine hydrolyzing) (EC 4.3.3.6)	pyridoxine biosynthesis protein
CAALFM_C401070WA	3635687	328	192	296	1656	1471	3186	272	2104	6.74	0.0000	0.0000	Up	Hgt17p	Hgt17p
CAALFM_C209590CA	3643800	298	409	454	2442	3555	2943	387	2980	6.70	0.0000	0.0000	Up	Cu/Pi carrier	Cu/Pi carrier
CAALFM_C700760CA	3637960	38	45	36	274	335	307	40	305	6.63	0.0000	0.0000	Up	Uncharacterized membrane protein	uncharacterized protein CAALFM_C700760CA

Down-regulated															
**Locus Tag**	**EntrezID**	**FLC1**	**FLC2**	**FLC3**	**FLC+AC1**	**FLC+AC2**	**FLC+AC3**	**FLC(mean)**	**FLC+AC(mean)**	**Fold Change**	**Pvalue**	**Padj**	**Sig**	**Description (Uniprot)**	**Description (NCBI)**
CAALFM_C107030CA	3639534	1011	1217	901	54	73	54	1043	60	-16.38	0.0000	0.0000	Down	Secreted protein RBT4 (PRY family protein 4) (Repressed by TUP1 protein 4)	Rbt4p

CAALFM_C403570WA	3645372	120	150	91	3	17	4	120	8	-14.00	0.0000	0.0000	Down	Hyphal wall protein 1 (Cell elongation protein 2)	Hwp1p
CAALFM_C703560WA	3646236	542	446	303	36	34	37	430	36	-10.94	0.0000	0.0000	Down	Virulence factor CaO19.6688	uncharacterized protein CAALFM_C703560WA
CAALFM_C401010CA	3635607	9944	13432	8707	1183	1050	1266	10694	1166	-8.17	0.0000	0.0000	Down	Dag7p	Dag7p
CAALFM_C404320WA	3646841	1235	1442	916	148	267	158	1198	191	-5.27	0.0000	0.0000	Down	Fre10p	Fre10p
CAALFM_CR08880CA	3641878	1582	2057	1857	308	298	299	1832	302	-5.07	0.0000	0.0000	Down	Uncharacterized protein	uncharacterized protein CAALFM_CR08880CA
CAALFM_C604610CA	3645969	1159	1046	779	179	155	160	995	165	-5.03	0.0000	0.0000	Down	Major facilitator superfamily multidrug transporter NAG3 (Multidrug resistance protein 97) (N-acetylglucosamine utilization protein 3) (Transmembrane protein 1)	Nag3p
CAALFM_C306760WA	3638502	248	2570	2433	461	486	391	2496	446	-4.60	0.0000	0.0000	Down	Uncharacterized protein	uncharacterized protein CAALFM_C30676

		5													0WA
CAALFM_C113940WA	3636185	2114	27190	22914	4557	4928	3887	23739	4457	-4.33	0.0000	0.0000	Down	Secreted beta-glucosidase SIM1 (EC 3.2.1.-)	SUN family protein
CAALFM_C103230CA	3636811	7176	7506	6564	1511	1521	1293	7082	1442	-3.91	0.0000	0.0000	Down	Probable metalloprotease ARX1 (EC 3.-.-.-) (Associated with ribosomal export complex protein 1)	putative hydrolase
CAALFM_C405780CA	3641811	125	174	211	35	35	34	170	35	-3.86	0.0000	0.0000	Down	Cfl2p	Cfl2p
CAALFM_CR05660WA	3646688	4533	4641	4013	990	983	768	4396	914	-3.81	0.0000	0.0000	Down	Protein SDA1	Sda1p
CAALFM_C402610CA	3637835	173	230	195	28	52	45	199	42	-3.74	0.0000	0.0000	Down	Sulfate permease	sulfate permease
CAALFM_C109510WA	3634765	2790	2827	2410	586	680	526	2676	597	-3.48	0.0000	0.0000	Down	Ribosome biogenesis protein YTM1	Ytm1p
CAALFM_C111480	3645348	17	2133	1818	541	179	585	1896	435	-3.36	0.0001	0.0001	Down	Phosphate transporter	phosphate transporter

WA		38													
CAALFM_C306370CA	3642258	4611	4628	3957	1107	1071	889	4399	1022	-3.30	0.0000	0.0000	Down	Uncharacterized protein	uncharacterized protein CAALFM_C306370CA
CAALFM_C100350CA	3639294	8931	12188	10138	1848	3475	1980	10419	2434	-3.28	0.0000	0.0000	Down	Hmx1p	Hmx1p
CAALFM_C600440CA	3647131	2688	2525	2457	645	646	539	2557	610	-3.19	0.0000	0.0000	Down	Ferroxidase	ferroxidase
CAALFM_C304480CA	3635211	845	777	1192	272	171	229	938	224	-3.19	0.0000	0.0000	Down	Ras2p	Ras2p
CAALFM_C302020WA	3636592	3483	3862	3325	928	822	803	3557	851	-3.18	0.0000	0.0000	Down	Multiple RNA-binding domain-containing protein 1	RNA-binding ribosome biosynthesis protein

TABLE S4. The list for all the primes used in this study.

No	Primers	Sequence (5’→3’)
1	CAALFM_C600430CA-F	AGCCACAAAGGACCAGTTCC
2	CAALFM_C600430CA-R	TCGCCTAACCCTTTTGCACT
3	CAALFM_C700280WA-F	GCCACTTCTTCTTGGACCCA
4	CAALFM_C700280WA-R	TGCAAAGCTTGGATGGCAAC
5	CAALFM_C404050CA-F	ACAAGGCTGCTGAAACCAGT
6	CAALFM_C404050CA-R	AAACAGAGGCTTGAGCTGGG
7	CAALFM_C107030CA-F	CTGCTGCCATTTTTGCTGGT
8	CAALFM_C107030CA-R	TAGCAACGGTGGCAGTTTCA
9	CAALFM_C703560WA-F	TCTACAGCACCAACCACAGC
10	CAALFM_C703560WA-R	TCAATGGGATCCACAGACTTGG
11	CAALFM_C407530WA-F	AGCCACAAAGGACCAGTTCC
12	CAALFM_C407530WA-R	TCGCCTAACCCTTTTGCACT
13	RIB4-F	GAGCTGGTTTGATTGAAGGCA
14	RIB4-R	TAGTGGCCATTTCGACAGCA

15	FAD1-F	CCGAATGGCAAATACCAACCA
16	FAD1-R	ACTCTACCCAACCGCTCATT
17	RIB5-F	TAAAGTTGGTGTGGCCCCAG
18	RIB5-R	ATTGACTGGTGCACCGTGTT
19	RIB1-F	TGCTAGCAGCCACGGAATAC
20	RIB1-R	TGTTGACGGTTTCGGTGTCA
21	CAALFM_CR03740CA-F	TGAACTAGGAATCCCCACAGC
22	CAALFM_CR03740CA-R	TTTCGTCACATTGGGCTGTT
23	CAALFM_C112810WA-F	AGTCACCACCACTAACAATGGA
24	CAALFM_C112810WA-R	GTGTACCTGGTTGAGCTGCT
25	PR26-F	GCCACCAGAAGCTGACTCTT
26	PR26-R	CTAGACCACCAACATCGGCA
27	RPN8-F	TGGCTCTGCTGCTGAAAAGA
28	RPN8-R	CCAGCAGCTTGGTCTCTGAT
29	PRE5-F	ACAGGGGCACACCTTTTTGA
30	PRE5-R	TGTACGAGCTGCTTGGGATC
31	PRE3-F	CGTTCTGGATCAGCAGCAGA

32	PRE3-R	GGAGTTTCACCAGGAGGCAA
33	PRE2-F	GTTGATTCTCGTGCCACAGC
34	PRE2-R	GCACCACCAGCCATAGTACC
35	POP4-F	GCGATTAACGGCTGCTGATT
36	POP4-R	ACAACAATTCCTCTGGTGCCT
37	UTP5-F	ACGAGTGCTAAAGGTGACCG
38	UTP5-R	ACAGACCCAAAAAGACTGCCT
39	NAN1-F	GCAAGGTCTCAGCAAACTGC
40	NAN1-R	AGCAACGGCATCTGATGACA
41	RIX7-F	CTACCGCAGACGAAGAGCAT
42	RIX7-R	AAGCCAACACCTGGGAAACA
43	MPP10-F	AGCCACAGCCAAAGGAAAGT
44	MPP10-R	TTCTGCTTCCAACCTGGCAA
45	18S rDNA-F	TCTTTCTTGATTTTGTGGGTGG
46	18S rDNA-R	TCGATAGTCCCTCTAAGAAGTG
